# Mod(mdg4) variants repress telomeric retrotransposon *HeT-A* by blocking subtelomeric enhancers

**DOI:** 10.1093/nar/gkac1034

**Published:** 2022-11-14

**Authors:** Chikara Takeuchi, Moe Yokoshi, Shu Kondo, Aoi Shibuya, Kuniaki Saito, Takashi Fukaya, Haruhiko Siomi, Yuka W Iwasaki

**Affiliations:** Department of Molecular Biology, Keio University School of Medicine, Tokyo 160-8582, Japan; Laboratory of Transcription Dynamics, Research Center for Biological Visualization, Institute for Quantitative Biosciences, The University of Tokyo, Tokyo 113-0032, Japan; Department of Chromosome Science, National Institute of Genetics, Research Organization of Information and Systems (ROIS), Shizuoka 411-8540, Japan; Department of Molecular Biology, Keio University School of Medicine, Tokyo 160-8582, Japan; Department of Chromosome Science, National Institute of Genetics, Research Organization of Information and Systems (ROIS), Shizuoka 411-8540, Japan; Laboratory of Transcription Dynamics, Research Center for Biological Visualization, Institute for Quantitative Biosciences, The University of Tokyo, Tokyo 113-0032, Japan; Department of Life Sciences, Graduate School of Arts and Sciences, The University of Tokyo, Tokyo 113-0032, Japan; Department of Molecular Biology, Keio University School of Medicine, Tokyo 160-8582, Japan; Department of Molecular Biology, Keio University School of Medicine, Tokyo 160-8582, Japan; Laboratory for Functional Non-coding Genomics, RIKEN Center for Integrative Medical Sciences, Yokohama 230-0045, Japan; Japan Science and Technology Agency (JST), Precursory Research for Embryonic Science and Technology (PRESTO), Saitama 332-0012, Japan

## Abstract

Telomeres in *Drosophila* are composed of sequential non-LTR retrotransposons *HeT-A*, *TART* and *TAHRE*. Although they are repressed by the PIWI-piRNA pathway or heterochromatin in the germline, the regulation of these retrotransposons in somatic cells is poorly understood. In this study, we demonstrated that specific splice variants of Mod(mdg4) repress *HeT-A* by blocking subtelomeric enhancers in ovarian somatic cells. Among the variants, we found that the Mod(mdg4)-N variant represses *HeT-A* expression the most efficiently. Subtelomeric sequences bound by Mod(mdg4)-N block enhancer activity within subtelomeric TAS-R repeats. This enhancer-blocking activity is increased by the tandem association of Mod(mdg4)-N to repetitive subtelomeric sequences. In addition, the association of Mod(mdg4)-N couples with the recruitment of RNA polymerase II to the subtelomeres, which reinforces its enhancer-blocking function. Our findings provide novel insights into how telomeric retrotransposons are regulated by the specific variants of insulator proteins associated with subtelomeric sequences.

## INTRODUCTION

Telomeres are specialized structures that protect the ends of chromosomes, and their loss of function can lead to various defects, including the fusion of chromosome ends (1). In mammals, telomere sequences are generally composed of short tandem repeats, such as TTAGGG, which are elongated by telomerase (2). Telomere elongation and terminal protection are controlled by multiple mechanisms. For example, the heterochromatin machinery (3), end-capping proteins (4–9) or telomeric repeating-containing RNA (TERRA) (10) play a critical role in telomeric regulation. Another important regulatory component is the subtelomere, which is a sequence neighboring telomeres. Subtelomeres are vital for telomere regulation, e.g. serving as transcription start sites of non-coding RNA, which are essential for telomere maintenance (10). Subtelomeres can also act as chromatin insulators, i.e. sequences that possess enhancer-blocking activity and act as epigenetic modification boundaries involved in the regulation of gene expression (11). In mammals, CTCF binds to subtelomeres and regulates the transcription of TERRA, which is essential for telomeric protection (12–14). However, it is unknown whether insulator-based telomeric control exists in diverse species.

**Figure 1.**
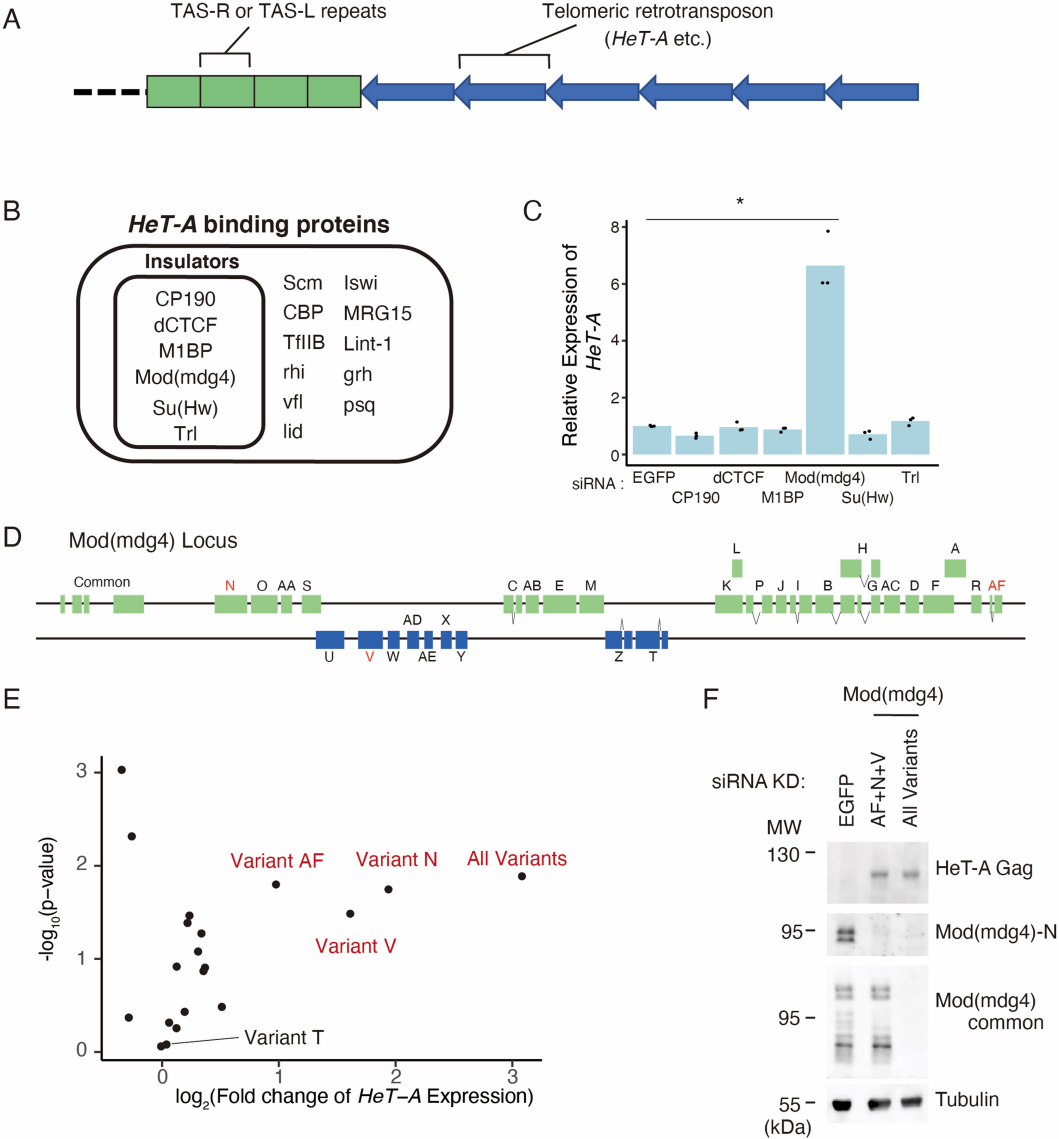
Mod(mdg4) restricts *HeT-A* expression in a variant-specific manner. (**A**) Schematic representation of a typical *D. melanogaster* telomere/subtelomere structure. On the right arms of chromosomes 2 and 3, the subtelomeres contain several TAS-R sequences and on the left arms of chromosomes 2 and 3, the subtelomeres contain several TAS-L sequences. Next to these subtelomeric TAS-R/L repeats, telomeric retrotransposons, such as *HeT-A* are connected unidirectionally. (**B**) Protein list with significant ChIP-seq peaks on *HeT-A* as revealed by the ChIP-Atlas (Oki *et al.*, 2018). Insulator proteins are highlighted by an inner rectangle. (**C**) siRNA knockdown (KD) screening for insulator genes in OSC, followed by qRT-PCR of *HeT-A* transcript expression levels. Values on the y-axis were normalized to *RP49* (*n* = 3). Each dot indicates the value obtained from different experiments. Asterisks indicate *P* < 0.05 in the two-sided *t*-test. (**D**) Schematic diagram of Mod(mdg4) locus. Mod(mdg4) has four common exons and 31 variant-specific exons. The names of the variants are indicated on the exons. Variants of interest are highlighted in red. (**E**) Volcano plot showing *HeT-A* expression level measured by qRT-PCR and the corresponding significance upon KD using siRNA targeting 19 Mod(mdg4) variants and all variants. Three variants (variant N, V, and AF) that upregulate *HeT-A* upon KD are labeled. Each dot represents the KD of the different variants (*n* = 3). The x-axis is the log_2_ ratio, and the y-axis is the log_10_ ratio. (**F**) Western blotting showing protein levels of tubulin (loading control), total Mod(mdg4), Mod(mdg4)-N, and *HeT-A* Gag upon the KD of EGFP (control), Mod(mdg4)-AF + N + V, or all Mod(mdg4) variants. The molecular weight (MW) is indicated on the left side of each image.

In contrast with these telomere maintenance mechanisms, some invertebrate species lack telomerase activity, and telomeres are replaced by retrotransposons: *TRAS* and *SART* in *Bombyx mori* (15,16) and *HeT-A*, *TART*, and *TAHRE* in *Drosophila melanogaster* (17–20). Telomeres of *D. melanogaster* lack tandem repeat sequences completely, and it has been proposed that telomere length depends on the transpositions of these retrotransposons (20–22) (Figure 1A). *HeT-A*, *TART* and *TAHRE* have notable features: (i) they exist only at the telomeres, (ii) they line up unidirectionally and (iii) *HeT-A* is the most abundant among these three telomeric retrotransposons. *HeT-A* contains Gag protein but not Pol protein, which includes reverse transcriptase; therefore, *HeT-A* uses Pol from the other telomeric transposons upon its transposition (21,22). These processes are regulated by multiple fast-evolving telomere-capping proteins (23–28). The subtelomere sequences of *D. melanogaster* also have unique features. These subtelomere sequences are composed of TAS-L or TAS-R repeats (29,30) (Figure 1A). Although the TAS-L monomer is composed of a non-coding sequence, TAS-R is highly similar to the Invader4 long terminal repeat (LTR) element (31,32), suggesting an additional relationship between retrotransposons and telomere/subtelomere evolution in this organism.

These unique features of telomere maintenance by retrotransposons in *D. melanogaster* resemble a symbiosis between the host and retrotransposon (20). However, de-repression of *HeT-A* retrotransposon in germ cells causes severe embryo abnormalities (33,34), and a recent genomic analysis of other *Drosophila* species has suggested that these retrotransposons evolve rapidly and replicate their copy number selfishly rather than symbiotically (21,26,35). Therefore, the host must regulate the telomeric retrotransposon activity. Multiple mechanisms repress telomeric retrotransposons (36). For example, the PIWI-piRNA pathway is a small RNA-mediated RNA-silencing mechanism (37) known to repress transposons, including telomeric retrotransposons in fly germline cells. The dysfunction of this pathway leads to strong *HeT-A* expression (38–41). The heterochromatin machinery also plays a critical role in regulating telomeric retrotransposons. The mutation of heterochromatin protein 1 (HP1; also known as Su(var)205) causes telomere fusion and increases the expression of telomeric retrotransposons (42–45). Additionally, some transcription factors, such as *Woc, Stwl, Hmr* or *Lhr*, and poly-A tail regulation machinery contribute to *HeT-A* repression in the ovaries (33,43–46). Most of these repressive machineries function in a germline-specific manner (47), but the expression of telomeric transposons is also regulated within somatic cells. For example, the expression of *HeT-A* is limited in the proliferating brain cells of larvae in a cell cycle-specific manner (48,49), suggesting that the dynamic expression pattern of *HeT-A* also has functions in somatic cells. However, the regulatory mechanism of telomeric transposons in somatic cells remains to be elucidated.

In this study, we demonstrated that specific splice variants of Mod(mdg4), an insulator protein in *Drosophila*, act as repressors of telomeric *HeT-A* retrotransposons by blocking subtelomeric enhancer activity in ovarian somatic cells (OSCs). Using RNAi knockdown (KD) screening, we demonstrated that the Mod(mdg4) variants N, V and AF (hereafter, Mod(mdg4)-N, V, AF, respectively), which are variants of Mod(mdg4) insulators with unknown functions, are responsible for *HeT-A* repression. Among these variants, Mod(mdg4)-N homozygous mutant flies display *HeT-A* de-repression in the ovary and a female sterility phenotype. Further analysis revealed that each Mod(mdg4) variant has binding specificity, and Mod(mdg4)-N is bound to both subtelomeric and telomeric loci, in addition to genome-wide binding sites. A live-imaging analysis demonstrated strong enhancer-blocking activity of the subtelomeric TAS-R sequences and weak enhancer-blocking activity of Mod(mdg4)-N-binding *HeT-A* sequences. These results indicate that Mod(mdg4)-N represses *HeT-A* expression by blocking subtelomeric enhancers. Further investigation into how Mod(mdg4) blocks the enhancer activities at TAS-R revealed that the important factors for inducing a strong enhancer blocking effect, specifically at the TAS-R regions, are the Pol II association site within TAS-R, capable of recruiting Pol II upon Mod(mdg4)-N association, and the highly repetitive nature of TAS-R sequences. Our findings provide novel insights into how insulator proteins mediate the link between subtelomeres and telomeres.

## MATERIALS AND METHODS

### List of *Het-A* binding proteins

The list of *HeT-A* binding proteins (the names of the ChIP antigens) was collected from the ChIP-Atlas (50,51). Using the dm6 datasets, we downloaded datasets with the following parameters: ‘ChIP: TFs and others’ for Experiment type, ‘All cell type’ for Cell type Class and ‘500’ for the threshold for significance. With this dataset, the peaks that overlapped with full-length *HeT-A* (chr2R:25261552–25268656) were extracted using bedtools (https://github.com/arq5x/bedtools2).

### Cell lines and culture condition

Ovarian somatic cells (OSC) were cultured as described (52,53). Briefly, OSC were cultured in Shield and Sang M3 Insect Medium (Sigma) supplemented with 10% fly extract, 10% fetal bovine serum, 0.6 mg/ml glutathione, and 10 μg/ml insulin. OSC were passed every second day.

### Fly strains

New alleles of mod(mdg4) were generated using the transgenic Cas9 system as previously described (54). The 20-bp sequence of the gRNA targeting the RN variant-specific exon of mod(mdg4) was as follows: GTAGTTGCGGAACACCAGCT. Multiple candidate mutant lines were established from individual male offspring of parents carrying both the nos-Cas9 and U6-gRNA transgenes. PCR was performed on their genomic DNA to amplify a region surrounding the gRNA target and their sequences were determined to search for indel mutations. Two lines, RN1-2 and RN1-4, were found to carry distinct frameshift mutations and were subjected to further functional analysis.

In all live-imaging experiments, *D. melanogaster* embryos at nuclear cycle 14 were analyzed. The following fly lines were used in this study: *nanos> MCP-GFP, His2Av-mRFP/CyO* (55), *DSCP_WT_-MS2-yellow-sna shadow enhancer* (55), *DSCP_WT_-MS2-yellow-gypsy-sna shadow enhancer* (56), *DSCP_WT_-MS2-yellow-HeT-A-sna shadow enhancer* (this study), *DSCP_WT_-MS2-yellow-HETRP-sna shadow enhancer* (this study), *DSCP_WT_-MS2-yellow-HeT-A-sna shadow enhancer*-*HeT-A* (this study), *DSCP_WT_-MS2-yellow-HETRP-sna shadow enhancer-HETRP* (this study).

### Cell transfection

For transfection of small interfering RNA (siRNA), 200 pmol siRNA duplex was transfected using the Cell Line 96‐well Nucleofector Kit SF (Lonza) and program DG150 of the 96‐well Shuttle Device (Lonza). For transfection of expression vectors, expression vectors were transfected using Xfect Transfection Reagent (TaKaRa Clontech), in accordance with the manufacturer's instructions. All siRNA sequences used in this study are listed in Supplementary Table S1.

### Generation of antibodies

The 1469–1901 bp region (for Mod(mdg4)-N antibody) or the 543–1364 bp region (for Mod(mdg4) common region antibody) of Mod(mdg4) variant N mRNA (Flybase: FBtr0084073) and the 3984–4511 bp region of HeT-A 23Zn-1(GenBank: U06920.2) were subcloned into pMAL-c2G and pGEX-5X-1 from OSC or ovary cDNA. These vectors were expressed in Rosseta-gami B(DE3) (Novagen) with 1 mM IPTG at 18°C overnight. MBP-tagged proteins and GST-tagged protein were purified with Amylose Resin (New England Biolabs) and Glutathione Sepharose 4B (Cytiva). These purified proteins were used as antigens, and hybridomas with SP2/O were generated and screened according to standard protocol (57).

### Quantitative reverse-transcription PCR

For OSC, RNA extraction and cDNA preparation were performed with SuperPrep II Cell Lysis & RT kit for qPCR from 1.0 × 10^5^ OSC. For fly ovaries, total RNA extraction was performed with ISOGEN II (Nippon Gene: 311-07361) and cDNA was synthesized with Transcription First Strand cDNA Synthesis Kit (Roche 04379012001). TB Green Premix Ex Taq II (Clontech) was used for quantitative PCR with indicated qPCR primers (Supplementary Table S1).

### Western blot

Samples were lysed to the concentration of 5.0 × 10^5^ OSC/1 ovary per 10 μl 1× Laemmli buffer and heated at 95°C for 5 min. Proteins were separated by SDS–polyacrylamide gel electrophoresis (PAGE) and transferred to a 0.45 μm nitrocellulose membrane (Cytiva). Membrane was washed by PBS and blocked with 3% skim milk in PBS-T, then incubated with primary antibody for 1 h at room temperature. For primary antibody, anti-beta tubulin (DSHB, E7) (1:5000), anti-c-Myc (DSHB, 9E10) (1:1000), anti-Ty1 antibody (Diagenode, C15200054) (1:2000), anti-HeT-A Gag supernatant (this study) (1:1), anti-Mod(mdg4) common region supernatant (this study) (1:4) and anti-Mod(mdg4) variant N supernatant (this study) (1:1) were used with indicated dilutions. After three washes with PBS-T, membrane was incubated with HRP-conjugated secondary antibody (MP Bioscience, 0855558) (1:5000) for 30 min, followed by three PBS-T washes. The membranes were incubated at room temperature for 1 h. The membrane was incubated with ECL Prime Western Blotting Detection Regent (Cytiva) and was exposed to Amersham Hyperfilm ECL (Cytiva). Exposed film was developed by X-ray film developer (KONICAMINOLTA TCX-101).

### Immunofluorescence

Ovaries were fixed with 4% paraformaldehyde for 20 min and washed once by PBS-T (0.2% Tween20 in PBS), followed by blocking by PBS-BT (0.2% Tween20 and 10 mg/ml BSA in PBS). Primary antibodies were diluted with PBS-BT and incubated with samples overnight at 4°C. For primary antibodies, anti-Orb (DSHB, 4H8) (1:200) was used with indicated dilutions. Following three washes with PBS-T, secondary fluorophore-conjugated antibody (ThermoFisher, A32727) was diluted at 1:1000 in PBS-BT and incubated with samples for 2 h at room temperature. The stained samples were washed three times with PBS-T and mounted by DAPI-containing medium. Samples were imaged with FV3000 (Olympus) confocal microscope.

### RNA fluorescence in situ hybridization (FISH)

*HeT-A* RNA fluorescence in situ hybridization (FISH) was performed on the ovaries as described previously (58) and according to the manufacturer's instructions using Quasar-labeled 670 Stellaris oligo probes (Supplemental Table 4). Briefly, the ovaries were subjected to FISH with 4% PFA and dissected into ovarioles. The ovarioles were incubated overnight with 100% methanol for penetration. The methanol was then gradually replaced with PBS-T (0.2% Tween20 in PBS). The ovarioles were fixed again with 4% PFA followed by 1 μg/ml proteinase K for 13 min. The proteinase K was then quenched twice with 2 mg/ml glycine. The ovarioles were washed twice with 1 ml of PBS-T and hybridized with FISH probes for 16 h at 37°C. The ovarioles were washed twice with wash buffer A, PBS and 1 μg/ml DAPI-containing PBS. The samples were imaged using an FV3000 confocal microscope (Olympus).

### Isolation of Ty1-Mod(mdg4) variants stable line OSC

Stable cell lines were established as described previously (59). In short, Mod(mdg4) variants were cloned into pPB-2 × Ty1-Tjen-EGFP-P2A-BlastR. Stable clones were selected by medium containing 50 μg/ml blasticidin.

### Plasmid construction

#### HeT-A/HETRP sequence cloning

*HeT-A* or HETRP sequences were cloned from the OSC genome. These sequences were amplified by PCR using specific primers (rep_HeT-A_F/R or rep_HETRP_F/R), and cloned to pBlueScript SK(+) EcoRI/NotI treated fragment by using NEBuilder HiFi DNA Assembly Master Mix (NEB). These plasmids are named pBS-HeT-A or pBS-HETRP respectively. Primer sequences for cloning are shown in Supplementary Table S1, and whole sequences of fragments are shown in Supplementary Table S2.

#### DSCP_WT_-MS2-yellow-HeT-A-sna shadow enhancer

A DNA fragment containing *HeT-A* sequence was amplified from pBS-HeT-A using primers (5′-ACA TGA AGC TTC TTC TCC GTT CTA CCT CAA T-3′) and (5′-ACG GGA AGC TTC ACA GGG TGC CGC AAA AAT TG-3′) and digested with HindIII. The resulting fragment was inserted into the unique HindIII site of pbphi-DSCP-MS2-yellow*-*sna shadow enhancer (55).

#### DSCP_WT_-MS2-yellow-HETRP-sna shadow enhancer

A DNA fragment containing HETRP sequence was amplified from pBS-HETRP using primers (5′-ACG GGA AGC TTG TGT GTC ATC CAT TTC GTT T-3′) and (5′-ACA TGA AGC TTC GAC GCG TAC ACA TAT TTC G -3′) and digested with HindIII. The resulting fragment was inserted into the unique HindIII site of pbphi-DSCP-MS2-yellow*-*sna shadow enhancer (55).

#### *DSCP_WT_-MS2-yellow-HeT-A-sna shadow enhancer*-*HeT-A*

A DNA fragment containing *HeT-A* sequence was amplified from pBS-HeT-A using primers (5′-ACA TGG CTA GCC TTC TCC GTT CTA CCT CAA TAT ATC-3′) and (5′- TTA AAG CTA GCC ACA GGG TGC CGC AAA AAT TG-3′) and digested with NheI. The resulting fragment was inserted into the unique NheI site of *DSCP_WT_-MS2-yellow-HeT-A-sna shadow enhancer*.

#### DSCP_WT_-MS2-yellow-HETRP-sna shadow enhancer-HETRP

A DNA fragment containing *HeT-A* sequence was amplified from pBS-HETRP using primers (5′-ACA TGG CTA GCG TGT GTC ATC CAT TTC GTT TAT TC-3′) and (5′- TTA AAG CTA GCC GAC GCG TAC ACA TAT TTC GC -3′) and digested with NheI. The resulting fragment was inserted into the unique NheI site of *DSCP_WT_-MS2-yellow-HETRP-sna shadow enhancer*.

### Site-specific transgenesis by phiC31 system

All reporter plasmids were integrated into a unique landing site on the third chromosome using the VK00033 strain (60). PhiC31 was maternally provided using the *vas-phiC31* strain (61). Microinjection was performed as previously described (62). In brief, 0–1 h embryos were collected and dechorionated with bleach. Aligned embryos were dried with silica gel for ∼7 min and covered with FL-100-1000CS silicone oil (Shin-Etsu Silicone). Subsequently, microinjection was performed using FemtoJet (Eppendorf) and DM IL LED inverted microscope (Leica) equipped with M-152 Micromanipulator (Narishige). Injection mixture typically contains ∼500 ng/μl plasmid DNA, 5 mM KCl, 0.1 mM phosphate buffer, pH 6.8. The mini-white marker was used for screening.

### MS2 live-imaging

Virgin females of *nanos> MCP-GFP, His2Av-mRFP/CyO* (55) were mated with males carrying the MS2 allele. The resulting embryos were dechorionated and mounted between a polyethylene membrane (Ube Film) and a coverslip (18 mm x 18 mm), and embedded in FL-100-450CS (Shin-Etsu Silicone). Embryos were imaged using LSM900 (Zeiss). Temperature was kept between 23.5 and 24.5°C during imaging. Plan-Apochromat 40×/1.4 N.A. oil immersion objective was used. A stack of 26 images separated by 0.5 μm was acquired at each time point, and the final time resolution was 16.8 sec/frame. Images were captured in 16-bit. Images were typically taken from the end of nuclear cycle 13 to the onset of gastrulation at nuclear cycle 14. During imaging, data acquisition was occasionally stopped for a few seconds to correct z-position, and data were concatenated afterwards. For each cross, three biological replicates were taken. The same laser power and microscope setting were used for each set of experiments. The laser power was measured using X-Cite XR2100 power meter (Lumen Dynamics).

### mRNA-seq

Total RNAs were isolated with the Isogen II reagent (Nippon Gene) according to the manufacturer's protocol. Poly(A)+ RNAs were obtained using Oligo-dT beads. Libraries were prepared with Illumina TruSeq stranded mRNA kit according to the manufacturer's protocol.

### Chromatin immunoprecipitation (ChIP)

ChIP experiments of Ty1-tagged Mod(mdg4) variants were performed using the truChIP Chromatin Shearing Kit according to the manufacturer's instructions with minor modifications. 2 × 10^7^ OSC were fixed with 1% formaldehyde for 10 min and quenched and lysed with buffers from the truChIP Chromatin Shearing Kit. Fixed chromatin was sheared with Bioruptor II (BMbio BR2012A) for 20 cycles of 30 s ON/30 s OFF, high settings. Sheared chromatin was immunoprecipitated by antibody-conjugated Dynabeads protein G for 2 h. Immunoprecipitated chromatin was incubated with Proteinase K and RNase, and de-crosslinked at 65°C overnight.

For ChIP experiments of RNA polymerase II, 2 × 10^7^ OSC were fixed with 1% formaldehyde for 10 min and quenched with final 0.1 M glycine for 5 min. After washing twice with PBS, nuclei were isolated with 1 ml of swelling buffer (25 mM HEPES–KOH (pH 7.5), 1.5 mM MgCl_2_, 10 mM KCl, 0.1% NP-40, 1 mM DTT and 1x Protease Inhibitor). Isolated nuclei were lysed in 400 μl of sonication buffer (50 mM HEPES–KOH (pH 7.4), 140 mM NaCl, 1 mM EDTA, 1% Triton X-100, 0.1% Na-deoxycholate, 0.1% SDS and 1 × protease inhibitor) and were sheared with Bioruptor II (BMbio BR2012A) for 20 cycles of 30 s ON/30 s OFF, high settings. 3 μg of Pol II antibody were incubated with chromatin overnight (63). The next day, 20 μl of Dynabeads M-280 Sheep anti-Mouse IgG was added to the sample and incubated for 1 h. Beads were washed and treated with Proteinase K and RNase as described (64). DNA was purified with isopropanol precipitation using Pellet Paint NF Co-precipitant (Merck: 70748). Fragments from the ChIP experiment were sheared to ∼200 bases using Covaris S220. These were used for library preparation with the NEBNext Ultra II DNA Library Prep Kit for Illumina (NEB) following the manufacturer's protocol.

### Dual luciferase assay

For the plasmid construction, the Nluc-PEST sequence was cloned downstream of the *Tj* enhancer and *Drosophila* synthetic core promoter, and the TAS-R sequences were cloned at both sides of the *Tj* enhancers using standard cloning techniques (Supplemental Table 2).

For the transfection, 200 pmol siRNAs were transfected into 2.0 × 10^6^ OSCs as described above, and the cells were passaged into four wells of a 24-well plate. On the day after the siRNA transfection, 5 ng of each indicated plasmid was transfected into the OSCs using Xfect. After 24 h of plasmid transfection, dual-luciferase assays were performed according to the manufacturer's instructions (Promega). The luciferase activity in each well was measured using Cytation 5 (BioTek) with a 1-s integration time.

### Enhancer blocking reporter assay in OSC

Reporters were constructed from OSC_Reporter_UAS_traffic jam_BoxB_d2eGFP_t2a_Blast (65) and pAc5.1 vector in which Flag-mCherry:P2A:PuroR gene was cloned using the indicated primer to add an additional restriction site (Supplemental Table 1). Wild-type and mutant *HETRP* sequences were inserted into reporter with NruI or SalI restriction enzyme and NEBuilder HiFi DNA assembly (NEB). These reporters were transfected with pHsp70-Myc-PBase plasmid as described (59). Cells were selected with puromycin and fluorescence was measured with SH800Z (Sony). Normalized EGFP intensity is the original EGFP signal intensity divided by mCherry intensity, which allows normalization of the effect of the insertion copy number.

### Fertility assay

7–10 virgin female flies were collected 3–5 days before mating. Virgin female flies were mated with the indicated male and kept at 25°C overnight. The next day, mated flies were transferred onto grape juice plate and kept at 25°C without light. After 3 h, eggs were counted and kept at 25°C again. After 24 h, hatching eggs were counted.

### Image analysis

All the image processing methods and analysis were implemented in MATLAB (R2020a, MathWorks).

### Segmentation of nuclei

For each time point, maximum projection was obtained for all z-sections per image. His2Av-mRFP was used to segment nuclei. 512 × 512 maximum projection images were initially cropped to 300 × 430 to remove nuclei at the edge, and used for subsequent analysis. For nuclei segmentation, His2Av images were first blurred with Gaussian filter to generate smooth images. Pixels expressing intensity higher than 5% of the global maxima in the histogram of His2Av channel were removed. Processed images were converted into binary images using a custom threshold-adaptative segmentation algorithm. Threshold values were determined at each time frame by taking account of (i) histogram distribution of His2Av channel and (ii) the number and the size of resulting connected components. Boundaries of components were then modified to locate MS2 transcription dots inside of nearest nuclei. In brief, pixels with intensity twice larger than mean intensity of MS2 channel were considered as transcription dots, and new binary image was created for each time frame. The Euclidean distances between the centroid of binarized transcription dot and all boundaries of segmented nuclei were calculated. Boundary of the nucleus with the smallest Euclidean distance was modified in order to capture transcription dot within a nucleus. Centroids of connected components in nuclei segmentation channel were used to compute the Voronoi cells of the image. Resulting binary images were manually corrected using Fiji (https://fiji.sc).

### Tracking of nuclei

Nuclei tracking was done by finding the object with minimal movement across the frames of interest. For each nucleus in a given frame, Euclidean distances between the centroids of the nucleus in the current time frame and the nuclei in the previous time frame were determined. Nucleus with the minimum Euclidean distance was considered as a same lineage.

### Recording of MS2 signals

3D raw images with all z-sections of MCP-GFP channel were used to record MS2 fluorescence signals. Using segmented regions from max projected images of His2Av- mRFP channel, fluorescence intensities within each nucleus were extracted. 3D fluorescence values were assigned to the nearest segmented regions of projected images. Signals of MS2 transcription dots were determined by calculating an integral of fluorescence intensities around the brightest pixel within each nucleus using a 2D Gaussian fitting method as described below. (i) The xyz position of transcription site was determined as the brightest pixel in each nucleus. (ii) A 2D Gaussian fitting was performed in a 11 × 11 pixels region with a single z-plane centering the transcription site to estimate a fluorescent dot intensity and a local background. Fitting was performed with the following formula

$$\begin{equation*}I\left( {x,y} \right) = \alpha + {I_0}ex{p^{\left( { - \left( {\frac{{{{\left( {x - {x_0}} \right)}^2}}}{{2\sigma _x^2}} + \frac{{\left( {y - {y_0}} \right)}}{{2\sigma _y^2}}} \right)} \right)}}\end{equation*}$$

where *α* is the local background intensity, *I*_0_ is the amplitude of the peak fluorescence intensity, *x*_0_ and *y*_0_ are the center of the peak, *σ_x_* and *σ_y_* are the spreads of the fluorescent dot. (iii) The intensity of MS2 transcription dot was calculated as $2\pi {\sigma _x}{\sigma _y}{I_0}$ from fitting parameters as an estimated integral value after subtracting the local background (66). Subsequently, minimum MS2 intensities were determined for individual trajectories and subtracted to make the baseline zero.

### Detection of transcriptional bursting

A transcriptional burst was defined as a local change in fluorescence intensity. First, signal trajectories were smoothed by averaging within a window of 5 timeframes. When a nucleus had above-threshold transcription activity, burst was considered to be started. Burst was considered to be ended when the intensity dropped below 55% of the local peak value. When the burst duration was less than 5 timeframes, it was considered as a false-positive derived from detection noise. When the signal trace exhibited continuous decreasing at the beginning of burst detection, it was also not considered as a burst. Location of defined burst was then moved two timeframes afterwards to better capture the center of individual bursting event. The same method and threshold value were used for each set of experiments.

### Description of bursting properties

From each trajectory, number of bursts, amplitude and duration of each burst, and total integrated signal (output) produced by each nucleus were measured. To determine amplitude, the peak value during the burst was measured using trajectories after smoothing by averaging within a window of 5 timeframes. Duration was determined by measuring the length of each burst. Total RNA production was measured by taking the area under the raw trajectory. Amplitude and duration for each nucleus were determined by taking the average of all analyzed bursts in a single nucleus.

### ChIP-seq data analysis—mapping and peak call

Adaptors added by NEBNext Ultra II were cut by cutadapt (67) using the following parameters (cutadapt -j 12 -a AGATCGGAAGAGCACACGTCTGAACTCCAGTCAC-A AGATCGGAAGAGCGTCGTGTAGGGAAAGAGTGTAGATCTCGGTGGTCGCCGTATCATT -m 20). For mapping to the genome, subsequent reads were mapped to the dm6 by bowtie2 using the following parameters (bowtie2 -p 16 -N 1). Peak call was performed by MACS using the following parameter: for Mod(mdg4) variants (macs2 callpeak -f BAM -q 1e-15 -g dm) and for PolII (macs2 callpeak -f BAM -g dm –outdir ../peakcall). To note, peak call for Mod(mdg4) was performed with strict cutoff values to rule out ambiguous peaks and we confirmed almost no peak was detected in Ty1-tag ChIP for wildtype OSC dataset. Binding motif was found with MEME-ChIP (68) with standard options. Coverage tracks were visualized by pyGenomeTracks (69) with standard options.

### ChIP-seq data analysis—handling of Pol II ChIP-seq data

Pausing index was calculated as previously described (70). Pol II signal of both promoter region (TSS-250∼TSS + 250) and gene body (TSS + 500∼TES-500) was calculated from a public dataset (71). Genes whose length is shorter than 1500 bp were removed from downstream analysis. To obtain the pausing index for each gene, we divided reads of promoter region by reads of gene body. Cumulative distribution plot was illustrated by Python. For identifying Pol II loss genes, maximum coverage was calculated for each Pol II peak. Fold change of this maximum value was calculated between EGFP KD and Mod(mdg4) variant N KD.

### mRNA-seq data analysis

The mRNA reads were mapped to dm6 by RSEM and STAR using the following parameters (rsem-calculate-expression –paired-end–p 8 –star –star-path ∼/anaconda3/bin –gzipped-read-file). The differentially expressed genes (DEGs) were detected using limma and edgeR. A gene ontology analysis was performed using DAVID (72). All the gene lists were used as the background.

For the transposon mapping, the paired-end reads were mapped with STAR with the-outFilterMultimapNmax 100 option, and first reads were extracted using samtools. Then, only the sense reads were counted with featureCounts with -M –fraction -s 2 options to exclude anti-sense transcripts. The statistical tests were performed with edgeR using the counts per million reads.

### Reanalysis of single-cell RNA-seq data

The data were obtained from Jevitt *et al.* (73). We used Cellranger (version 7.0.0) (74) to map the transcriptome. As a reference, we used the dm6 data with a custom annotation (GTF format) file. The GTF file was downloaded from the ensemble database and modified to distinguish between each Mod(mdg4) transcript. This GTF file was deposited in GitHub. After mapping, each UMI was annotated into cell-type clusters as reported in the original paper (73), and the transcripts were counted within each cluster using the original Python script. The clusters were combined into the following categories: germ cells (1. Germline cluster 1, 2. Germline cluster 2) and early follicle cells (7. The mitotic follicle cells (stg. 1–5), 8. Postmitotic follicle cells (stg. 6–8), 7. Vitellogenic MBFCs (g. 8) 1, 8. Vitellogenic MBFCs (g. 8) 2, 11. Vitellogenic MBFCs (g. 8) 3, 12. Vitellogenic MBFCs (g. 8) 4, 13. Vitellogenic MBFCs (g. 9–10A) 1, 14. Vitellogenic MBFCs (g. 9–10A) 2, 15. Vitellogenic MBFCs (g. 9–10A) 3, 16. The choriogenic MBFCs (g. 10B)), and late follicle cells (17. dorsal appendage-forming follicle cells, 18. The choriogenic MBFCs (g. 12), 19. The choriogenic MBFCs (g. 14) 1, 20. The choriogenic MBFCs (g. 14) and 2), and the RPM was calculated within each category.

## RESULTS

### Mod(mdg4) variants N, V and AF repress *HeT-A* expression independently of heterochromatin formation

*HeT-A*, the most abundant of the telomeric transposons in *Drosophila* (Figure 1A) (35), is repressed by the PIWI–piRNA pathway in *Drosophila* germline cells. This is due to the expression of piRNAs against *HeT-A*, processed from the Rhino-dependent transcription of piRNA precursor transcripts (75); therefore, *HeT-A* is dramatically upregulated in the ovaries of *piwi* mutant flies. In contrast, piRNAs against *HeT-A* are not present in the ovarian follicle cells and the cell line derived from follicle cells, named OSC (52,53), where only Rhino-independent PIWI–piRNA regulation occurs (53). A reanalysis of previously published RNA-seq data revealed that *HeT-A* up-regulation was not observed upon *Piwi* knockdown (KD) in the OSC (Supplementary Figure S1A) (76). The KD of linker histone H1, which is indispensable for heterochromatin maintenance, also did not cause the upregulation of *HeT-A* (Supplementary Figure S1A) (76–78) and the levels of the heterochromatin histone mark H3K9me3 on *HeT-A* were relatively low compared with heterochromatic transposons, including *mdg1*, in the OSC (Supplementary Figure S1B). These results suggested the existence of a piRNA- and heterochromatin-independent *HeT-A* repression mechanism in the OSC. To gain insight into the transcriptional regulation of the retrotransposons at the telomeres, we searched the ChIP-Atlas database, which contains previously published ChIP-seq data sets, for proteins associated with *HeT-A* sequences (50,51). We found that several insulator proteins were potentially associated with the *HeT-A* regions (Figure 1B, Materials and Methods). To test the possibility that *HeT-A* is regulated by these insulator proteins, we performed a KD screen using siRNAs. This revealed that Mod(mdg4) KD led to the de-repression of *HeT-A* retrotransposons in the OSC (Figure 1C).

The *Mod(mdg4)* locus produces as many as 31 variants owing to trans-splicing (Figure 1D) (79). Among the 31 variants, only variants H and T have been reported (80–84). Almost all the variants were expressed in the OSC at various levels (Supplementary Figure S1C). To determine which variants were responsible for the *HeT-A* repression, we performed a KD screen using variant-specific siRNAs. The KD of variants N, V, and AF [Mod(mdg4)-N, V, AF], all of which are variants with unknown functions, resulted in the upregulation of *HeT-A* expression levels (Figure 1E). We further performed western blotting using specific antibodies generated against Mod(mdg4)-N (Supplementary Figure S1D), a common exon of Mod(mdg4) (Supplementary Figure S1E) and HeT-A Gag (Supplementary Figure S1F). This revealed that, consistent with the RNA levels, the *HeT-A* Gag protein became detectable upon the KD of these three variants (N + V + AF) to a level close to that observed upon the KD of all the variants (Figure 1F, S2A–C). These results indicate that Mod(mdg4)-N, V, and AF repress *HeT-A* expression in a piRNA- and heterochromatin-independent manner.

### Mod(mdg4)-N mutant flies show elevated *HeT-A* expression

**Figure 2.**
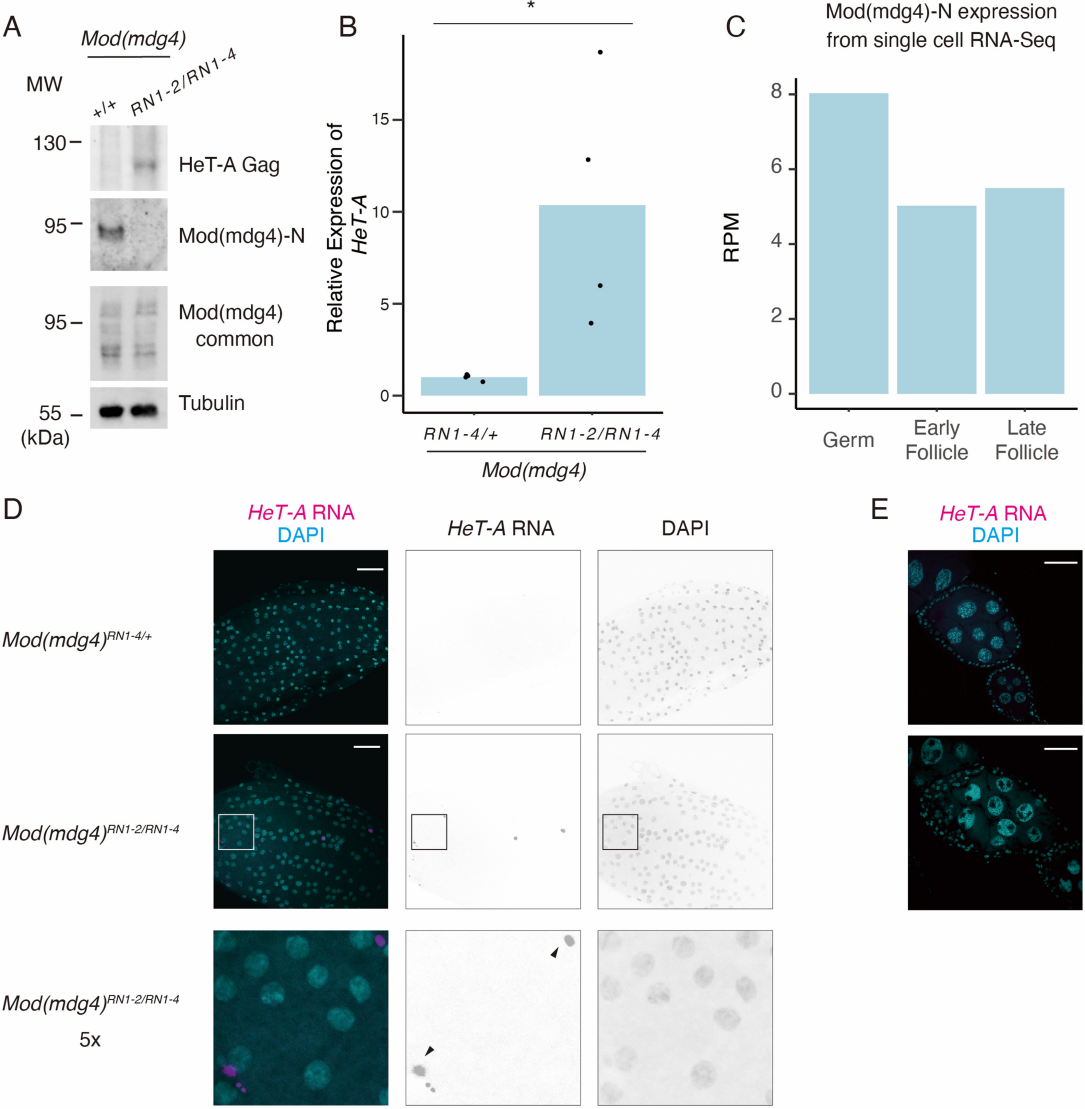
Mod(mdg4)-N plays a critical role in *HeT-A* repression in the follicle cells of the ovaries. (**A**) Western blots showing protein levels of *HeT-A* Gag, Mod(mdg4)-N, total Mod(mdg4), and tubulin (loading control) in ovaries of wild-type (+/+; OregonR) or trans-heterozygous Mod(mdg4)*^RN1-2/RN1-4^* mutants. The molecular weight (MW) is indicated on the left side of each image. (**B**) Bar plot showing *HeT-A* expression as measured by RT-qPCR. Values on the y-axis were normalized to *RP49* (n = 4). Each dot indicates the value obtained from different experiments. Asterisks indicate p < 0.05 in the two-sided *t*-test. (**C**) Bar plot showing Mod(mdg4)-N expression in germ cells, early follicles, and late follicle cells obtained from single-cell RNA-seq data from Jevitt *et al.* (73). The y-axis indicates the RPM for these cell types. (**D**) RNA-FISH images of stage 13–14 egg chambers. Images show DAPI staining (right), *HeT-A* RNA FISH (middle), and merging of the two (left). The bottom images are enlarged images of the Mod(mdg4)-N*^RN1-2/1^^–^^4^* mutants. Black arrows indicate *HeT-A* foci. (**E**) RNA-FISH images of stage 5–7 egg chambers. The egg chambers were stained with DAPI (cyan) and *HeT-A* RNA FISH (magenta).

The Mod(mdg4)-N KD resulted in the greatest degree of de-silencing of *HeT-A* expression among all the tested variants in the OSC (Figure 1E). To investigate whether *HeT-A* upregulation could be observed *in vivo* upon Mod(mdg4)-N loss and the physiological role of this specific variant, we generated two lines of Mod(mdg4)-N mutant flies (*RN1-2* and *RN1-4*) that harbor nucleotide deletions at the variant-specific exon using CRISPR-Cas9 (54) (Supplementary Figure S3A). Both deletions caused a frameshift in the coding sequence of the Mod(mdg4)-N-specific exon, and the Mod(mdg4)-N protein was eliminated completely in the homozygous or trans-heterozygous mutant flies (Figure 2A, S2D–F, S3B). In these homozygous or trans-heterozygous mutant ovaries, we observed increased expression of both *HeT-A* transcripts and *HeT-A* Gag proteins (Figure 2A–B, S3B–C). This result indicates that Mod(mdg4)-N represses *HeT-A* expression *in vivo* as well as in the OSC.

To understand which cells in the ovary possess this Mod(mdg4)-N-mediated *HeT-A* regulation, we reanalyzed previously published single-cell RNA-seq (scRNA-seq) data from *Drosophila* ovaries (73). We selected cell clusters of three categories as defined in a previous publication: germ cells, early follicle cells, and late follicle cells. We searched for reads mapped specifically to the Mod(mdg4)-N variant. This revealed that Mod(mdg4)-N was expressed in both germ and follicle cells (Figure 2C). Despite the ubiquitous expression of Mod(mdg4)-N in the ovary, RNA-FISH of *HeT-A* transcripts revealed that *HeT-A* was de-repressed specifically in the late-stage follicle cells in the Mod(mdg4)-N mutant ovaries (Figure 2D, E). These results indicate that, although Mod(mdg4)-N is expressed in both germ and somatic cells, it is especially functional for *HeT-A* repression in somatic cells. In the germ cells, the PIWI–piRNA pathway is active; therefore, *HeT-A* is likely to be still silenced without regulation by Mod(mdg4)-N.

Interestingly, the flies that were homozygous for each mutant allele and the trans-heterozygous flies were viable yet exhibited female sterility (Supplementary Figure S3D). Although the mutants of previously described Mod(mdg4) variants (variants T or H) did not show such a female sterility phenotype, this phenotype resembled previously reported hypomorphic mutants in common regions of Mod(mdg4) (85). The expression levels of variants other than Mod(mdg4)-N were not affected in this mutant (Figure 2A, Supplementary Figure S3B), suggesting that Mod(mdg4)-N was responsible for the female sterility phenotype. Macroscopically, the ovaries of the trans-heterozygous mutants were not atrophic (Supplementary Figure S3E), indicating that ovary development was not drastically impaired. Histologically, although the tissue was partly distorted, Orb (oocyte marker)-positive cells were present in the ovarioles of the trans-heterozygous Mod(mdg4)-N mutants (Supplementary Figure S3F). However, we observed eggs in the ovaries of trans-heterozygous Mod(mdg4)-N mutants and follicle cells remaining in the egg chambers (Supplementary Figure S3G), suggesting ovulation defects in the Mod(mdg4)-N mutant flies.

To understand the basis of the ovulation defect in the Mod(mdg4)-N mutant, we performed an RNA-seq analysis of the mutant ovaries and detected 505 upregulated and 911 downregulated genes (Supplementary Figure S3H, Supplemental Table 3). We performed a gene ontology analysis with both upregulated and downregulated genes (72). This revealed that in the Mod(mdg4) mutant, the GO term ‘cytoplasmic translation’ was enriched for the downregulated genes (Supplementary Figure S3I), and a series of cytoplasmic ribosomal proteins were included in the list of downregulated genes (Supplemental Table 3). The loss of ribosomal proteins can cause defects in follicle cells (86,87); therefore, the misregulation of genes encoding ribosomal proteins caused by the loss of Mod(mdg4)-N may induce follicle cell rupture defects. Whether the misregulation of genes encoding ribosomal proteins is linked to *HeT-A* expression remains unclear. Altogether, these results indicate that Mod(mdg4)-N plays a vital role in *HeT-A* repression *in vivo* and female fertility in the ovary.

### Mod(mdg4)-N associates with telomeric and subtelomeric repeats

**Figure 3.**
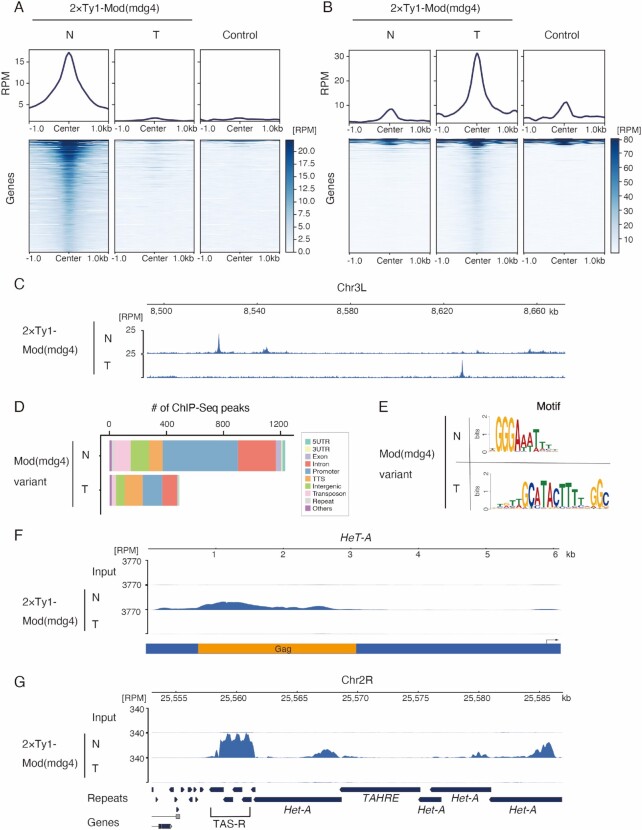
Mod(mdg4)-N associates with telomeric/subtelomeric regions in a variant-specific manner. (**A, B**) Metaplot and heatmap indicating Mod(mdg4)-N, -T, and control (anti-Ty1 ChIP against wild-type OSC) signal level within 1.0 kb of Mod(mdg4)-N (A) or -T (B) peaks. The heatmap is sorted by the intensity of the Mod(mdg4)-N (A) or -T (B) signals. The y-axes of the metaplots are normalized using the RPM. Note that some sites of the Mod(mdg4)-T peaks (B) display high background signals because Mod(mdg4)-T strongly accumulates on gypsy retrotransposons, which have many copies within the genome. (**C**) Distribution of Mod(mdg4) variant. Coverage panel showing ChIP-seq results for 2 × Ty1-tagged Mod(mdg4) variants (N, T) in the dm6 chr3L 8.50 Mb-8.66 Mb region. (**D**) Functional annotation of the peaks of Mod(mdg4)-N and -T identified using HOMER. The y-axis represents the number of peaks for each Mod(mdg4) variant. We used only highly confident (FDR < 1e–15) ChIP-seq peaks for the analysis to avoid ambiguous peaks. (**E**) Sequence logos showing clear enrichment around the ChIP-seq peaks of Mod(mdg4)-N and -T. These motifs were identified via de novo motif discovery analysis using MEME (Multiple Em for Motif Elicitation). (**F**) Coverage panel showing ChIP-seq of Mod(mdg4)-N and -T on the consensus sequence of *HeT-A* from repbase. Coverage of each ChIP-seq experiment is shown with blue. The y-axis is normalized with RPM. Below coverage panel of *HeT-A*, the reading frame of HeT-A Gag protein is shown in orange, and the black arrow indicates the *HeT-A* promoter. (**G**) Coverage panel showing ChIP-seq of Mod(mdg4)-N and -T near the subtelomere (TAS-R)/telomere region in dm6. The coverage of each ChIP-seq experiment is indicated in blue. The y-axis is normalized to RPM.

To determine how Mod(mdg4)-N represses *HeT-A* expression, we performed a ChIP-seq analysis by generating stable cell lines expressing Ty1-tagged Mod(mdg4) variants (Supplementary Figure S4A). The monoclonal antibody generated for Mod(mdg4)-N was unsuitable for ChIP experiments, so we performed ChIP-seq using the tagged Mod(mdg4) variants. The Ty1-tag was used because it lacks lysine, which is the primary target of formaldehyde, and has been successfully used for ChIP analyses (88). Mod(mdg4)-T, which forms protein complexes with Su(Hw) and CP190 as gypsy insulators and binds to the same location as Su(Hw) (89), was used as a control. To avoid artifacts caused by phantoms or pseudo-peaks (90), wild-type (WT) cells were also used to generate ChIP-seq libraries. Of the 31 Mod(mdg4) variants, 27 have FLYWCH domains in variant-specific regions (91). FLYWCH domains are C2H2-type zinc finger domains conserved in eukaryotes and are necessary for DNA binding or recruitment to chromatin (92,93), suggesting that the genomic binding patterns of the Mod(mdg4) variants may be different (85). Consistent with this view, the Mod(mdg4)-N and -T ChIP-seq peaks in the genome showed variant-specific chromatin localization patterns and did not overlap (Figure 3A–C), and the peaks were associated with genomic regions with different annotations (Figure 3D). Furthermore, a motif analysis using MEME revealed the sequence specificity of each Mod(mdg4) variant (Figure 3E, Supplementary Figure S4B). Consistent with a previous report (89), the Mod(mdg4)-T motif was highly similar to the previously identified Su(Hw) motif (Supplementary Figure S4C) (94), confirming that the results of the Ty1 tag-based ChIP-seq reflect the endogenous distribution of each Mod(mdg4) variant.

Next, we investigated how Mod(mdg4)-N is associated with telomere/subtelomere sequences. As expected from the KD screen (Figure 1E), mapping to the consensus sequence of *HeT-A* and reference genome revealed that Mod(mdg4)-N bound to *HeT-A*, whereas Mod(mdg4)-T did not (Figure 3F). Additionally, Mod(mdg4)-N was strongly enriched in the TAS-R repeats (Figure 3G), but not in the TAS-L repeats (Supplementary Figure S4D). Taken together, the accumulation of Mod(mdg4)-N in both the telomeric *HeT-A* and subtelomeric TAS-R repeats suggests that this variant regulates the expression of *HeT-A* by associating at these loci.

### The regulation of expression by Mod(mdg4)-N occurs specifically at *HeT-A*

**Figure 4.**
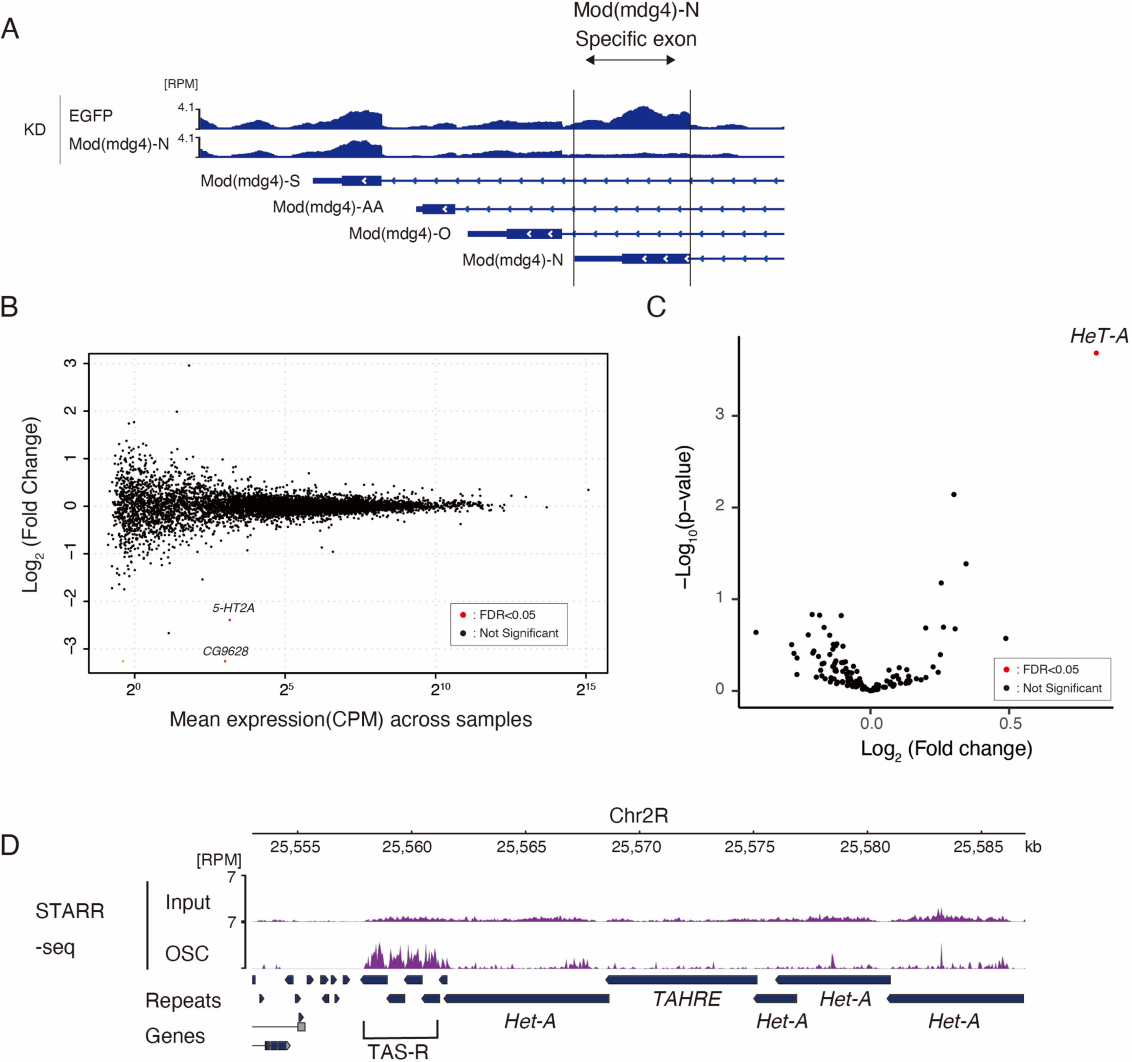
Specific regulation of *HeT-A* expression by Mod(mdg4)-N. (**A**) Coverage tracks of RNA-seq on EGFP KD or Mod(mdg4)-N KD at (mdg4)-locus (dm6 chr3R:21,370,172–21,373,552). The y-axis is normalized to RPM. The Mod(mdg4)-N-specific exon is highlighted with an arrow. Below the coverage, annotations of each Mod(mdg4) variant are indicated. (**B**) MA plot of TPM values (log_10_ scale) for mRNA in the Mod(mdg4)-N KD versus EGFP KD OSC (*n* = 2 for each condition). Differentially expressed genes (DEGs) are denoted by red dots. Note that Mod(mdg4) was not included in the DEGs because only Mod(mdg4) variant N was knocked down. (**C**) Volcano plot showing the differential expression of retrotransposons in Mod(mdg4)-N KD versus EGFP KD OSC. The x-axis is the log_2_ ratio, and the y-axis is the log_10_ ratio. Differentially expressed genes (DEGs) are denoted by red dots. (**D**) Coverage panel showing STARR-seq signal (purple) for OSC and its input near the subtelomere (TAS-R)/telomere region from dm6. The y-axis is normalized to RPM. These data are from the reanalysis of the STARR-seq results of Arnold *et al.* (2013).

Mod(mdg4)-N is associated with various genomic loci in OSCs (Figure 3). Therefore, we aimed to determine the extent to which Mod(mdg4)-N affects gene expression. To answer this question, we performed RNA-seq on Mod(mdg4)-N KD cells, which specifically depleted the -N isoform of Mod(mdg4) (Figure 4A). Unexpectedly, the expression of most protein-coding genes remained unchanged (Figure 4B). Only two genes were identified as differentially expressed genes (DEGs), despite the association of Mod(mdg4)-N at numerous gene bodies (331 peaks) and promoters (527 peaks) (Figure 3D). These two genes were *CG9628* and *5-HT2A*, whose biological functions are unrelated to telomere/subtelomere or transposon expression. In addition, when we analyzed the expression levels of transposons in the Mod(mdg4)-N KD cells, we found that the effect was limited to *HeT-A* (Figure 4C). These results show that Mod(mdg4)-N specifically regulates expression of *HeT-A*, despite its genome-wide association. Additionally, these results suggest that Mod(mdg4)-N does not directly regulate the expression of associated genes, but rather regulates expression in a context-specific manner.

### Subtelomeric TAS-R regions harbor enhancer activity

Since Mod(mdg4)-N cannot directly regulate most of its associated genes, we hypothesized that Mod(mdg4)-N has a specific function at telomeric regions. Previous reports have suggested the enhancer-blocking activity of Mod(mdg4)-T as a component of *gypsy* insulators (82,95). Given the strong binding of Mod(mdg4)-N to the telomeric and subtelomeric repeats, Mod(mdg4)-N may block enhancers located near telomeres, thereby repressing *HeT-A* expression. To search for enhancers near the telomeres, we reanalyzed STARR-seq [self-transcribing active regulatory region (STARR) sequencing] data obtained using OSCs (96). STARR-seq is a method for the genome-wide detection of enhancer activity by inserting randomly fragmented genomic sequences into reporter plasmids. By examining the regulation levels of the reporter gene, the enhancer activity of the randomly inserted fragments can be analyzed. The inserted fragments are deep-sequenced, and the genomic regions with accumulated reads indicate the regions with strong enhancer activity (96). Using this dataset, we analyzed the genomic regions near the telomeres and observed that TAS-R had strong enhancer activity in the OSC, whereas enhancer activity was not observed for *HeT-A* or TAS-L (Figure 4D, Supplementary Figure S5A, B). This result, together with the finding that Mod(mdg4)-N does not bind to TAS-L (Supplementary Figure S4D), indicates that different modes of regulation occur for the *HeT-A* associated with TAS-R and TAS-L. TAS-L does not possess enhancer activity, and therefore, the lack of Mod(mdg4)-N at TAS-L does not result in the de-repression of *HeT-A*. It is plausible that the TAS-R repeats possess enhancer activity because they are derived from the LTR sequences of the *Invader4* retrotransposon (31,32) and because LTR sequences generally have both enhancer and promoter activity (97). With these observations on the Mod(mdg4)-N-binding features and cis-regulatory element landscape, we hypothesized that Mod(mdg4)-N blocks subtelomeric enhancers at TAS-R to suppress *HeT-A* expression.

### Subtelomeric TAS-R repeats possess enhancer-blocking activity

**Figure 5.**
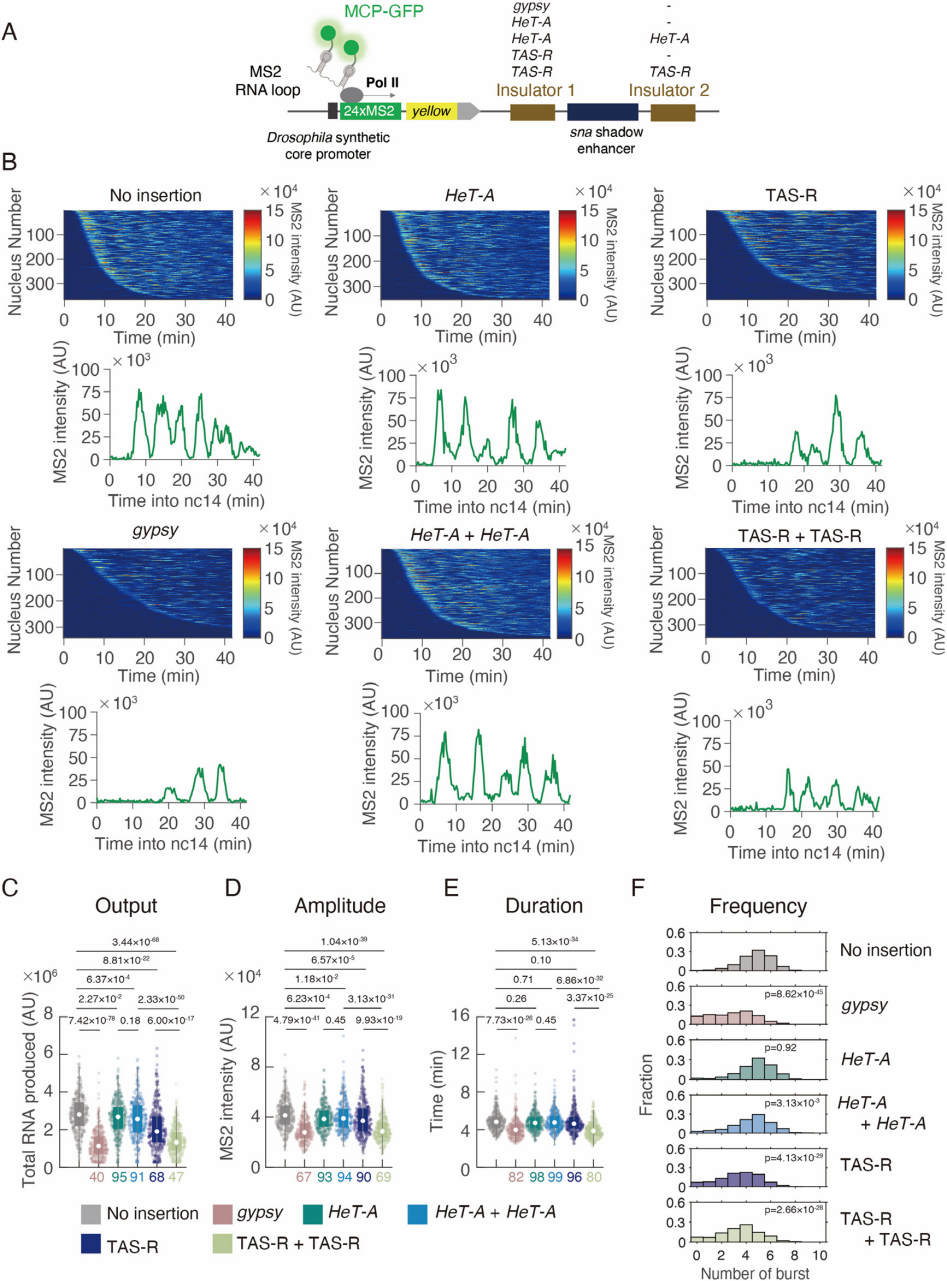
Subtelomeric TAS-R repeats possess enhancer-blocking activity. (**A**) Schematic representation of the yellow reporter gene containing the 155-bp *Drosophila* synthetic core promoter (DSCP), the 1.5-kb *sna* shadow enhancer, and 24 × MS2 RNA stem loops within the 5′ UTR. Insulator candidate sequences were inserted on both sides of the *sna* shadow enhancer. The expression of this reporter gene was visualized using MCP-GFP proteins binding to MS2 RNA stem loops. (**B**) (Upper) MS2 trajectories for all analyzed nuclei. Each row represents the MS2 trajectory for a single nucleus. A total of 365 (no insertion), 340 (gypsy), 352 (*HeT-A*), 365 (*HeT-A* + *HeT-A*), 365 (TAS-R), and 349 (TAS-R + TAS-R) ventral-most nuclei, respectively, were analyzed from three independent embryos for the reporter genes with no insertion of insulators, *gypsy* insulator, single insertion of *HeT-A*, double insertion of *HeT-A* (*HeT-A* + *HeT-A*), single insertion of TAS-R, or double insertion of TAS-R (TAS-R + TAS-R). Nuclei are ordered by their onset of transcription in the nuclear cycle 14. AU; arbitrary unit. (Lower) Representative trajectory of transcriptional activity of the MS2 reporter gene with no insertion of insulators, *gypsy* insulator, single insertion of *HeT-A*, double insertion of *HeT-A* (*HeT-A* + *HeT-A*), single insertion of TAS-R, or double insertion of TAS-R (TAS-R + TAS-R). (**C–E**) Boxplots showing the distribution of the total output (C), burst amplitude (D), and burst duration (E). The box indicates the lower (25%) and upper (75%) quantiles, and the open circle indicates the median. Whiskers extend to the most extreme non-outlier data points. A total of 365 (no insertion), 340 (gypsy), 352 (*HeT-A*), 365 (*HeT-A* + *HeT-A*), 365 (TAS-R), and 349 (TAS-R + TAS-R) ventral-most nuclei, respectively, were analyzed from three independent embryos for reporter genes with no insertion of insulators, *gypsy* insulator, single insertion of *HeT-A*, double insertion of *HeT-A* (*HeT-A* + *HeT-A*), single insertion of TAS-R, or double insertion of TAS-R (TAS-R + TAS-R) from left to right. The median values relative to the control reporter are shown at the bottom. The p-values of the two-sided Wilcoxon rank-sum test are shown at the top. AU; arbitrary unit. (**F**) Histograms showing the distribution of the burst frequency during the nuclear cycle 14 stage. A total of 365 (no insertion), 340 (gypsy), 352 (*HeT-A*), 365 (*HeT-A* + *HeT-A*), 365 (TAS-R), and 349 (TAS-R + TAS-R) ventral-most nuclei, respectively, were analyzed from three independent embryos for reporter genes with no insertion of insulators, *gypsy* insulator, single insertion of *HeT-A*, double insertion of *HeT-A* (*HeT-A* + *HeT-A*), single insertion of TAS-R, or double insertion of TAS-R (TAS-R + TAS-R) from top to bottom. The p-values of the two-sided Wilcoxon rank-sum test are shown within the windows.

We performed a live-imaging analysis of the enhancer-blocking activities of the Mod(mdg4)-N-binding sequences in *HeT-A* and TAS-R in living embryos, where Mod(mdg4) is highly expressed (85). In this imaging system, the *sna* shadow enhancer causes a transcriptional burst from the *Drosophila* synthetic core promoter (DSCP), a collection of core promoter motifs. The 24xMS2-yellow synthetic gene is transcribed from the DSCP under the control of the *sna* shadow enhancer. The MS2 coat protein (MCP) is a single-stranded RNA phage capsid protein that binds to the MS2 19-nucleotide RNA stem loop with high affinity (98), and the MCP-GFP fusion protein recognizes the MS2 repeats in this synthetic gene (Figure 5A, Supplemental Video 1–2). With this imaging system, the transcriptional dynamics and enhancer function can be precisely visualized (99–101). For the enhancer-blocking assay, we tested Mod(mdg4)-N-binding TAS-R (520 bp) and *HeT-A* (999 bp) sequences (Supplementary Figure S6, Supplemental Table 2). We compared the TAS-R and *HeT-A* sequences with the gypsy insulator sequence, which strongly inhibits enhancer-promoter interactions via the gypsy insulator proteins (101–103). Both *HeT-A* and TAS-R sequences decreased the total output of transcription although the effect of the *HeT-A* sequence was significantly weaker than that of the TAS-R sequence (Figure 5B, C: decreased to 95% and 68% compared with control). With the *HeT-A* sequence insertion, only amplitude per burst slightly decreased (Figure 5D–F). On the other hand, single insertion of TAS-R decreased both amplitude and frequency of the transcriptional burst (Figure 5D–F).

Considering that the Mod(mdg4)-N mutants displayed a 5–10-fold higher *HeT-A* expression in the ovary (Figure 2B), this enhancer-blocking activity of a single TAS-R or *HeT-A* was weaker than expected. Both TAS-R and *HeT-A* exist as tandem repeats in the *D. melanogaster* genome (20,29), and the pairing of insulators generally facilitates enhancer-blocking function (104). Therefore, we hypothesized that mimicking the *in vivo* tandem repeats of TAS-R and *HeT-A* may be important for assessing the enhancer-blocking activity of these sequences. To test this, we inserted TAS-R or *HeT-A* sequences on both sides of the *sna* shadow enhancer. With two copies of TAS-R (TAS-R + TAS-R), total output decreased considerably compared with wildtype and single insertion of TAS-R (Figure 5C), accompanied with significant decrease of both amplitude and duration of the transcriptional burst (Figure 5D, E). This result suggests that multiple repeats in the genome additively facilitate the enhancer-blocking activity of the TAS-R sequences. In contrast, two copies of *HeT-A* sequences bound by Mod(mdg4)-N (*HeT-A* + *HeT-A*) did not result in a significant difference in either the total output of transcription, amplitude, or duration of transcriptional burst, when compared to single *HeT-A* insertion (Figure 5C–E). These results indicate that Mod(mdg4)-N-binding TAS-R and *HeT-A* sequences are *bona fide* insulators while the effect of multiple repeats of insulator is specific to TAS-R sequences. Since enhancer-blocking function of *HeT-A* is weaker than that of TAS-R, and multiple repeats of TAS-R can result in stronger enhancer blocking activity, repetitive TAS-R has a major function in enhancer blocking activity, while *HeT-A* may assist that function.

To confirm the impact of the Mod(mdg4)-N-mediated enhancer-blocking function on TAS-R sequences, we performed luciferase assays upon loss of Mod(mdg4). Because the Mod(mdg4)-N mutant could not lay eggs and the Mod(mdg4) transcripts are maternally deposited (85), we used the OSC for further analysis. In this experiment, the TAS-R sequences were inserted on both sides of the *Tj* enhancer, and the downstream NanoLuc gene was transcribed from the DSCP. Firefly luciferase plasmids were used as internal controls (Supplementary Figure S7A). Using this reporter, we were able to determine that the insertion of the TAS-R repeats could regulate the expression of the NanoLuc reporter, consistent with live-imaging analysis (Supplementary Figure S7B). Furthermore, the KD of Mod(mdg4)-N resulted in de-silencing of the reporter activity (Supplementary Figure S7C, D), confirming that Mod(mdg4)-N was involved in the enhancer-blocking activity of TAS-R.

### RNA polymerase II is recruited to Mod(mdg4)-N binding sites

We further examined the molecular details of how Mod(mdg4)-N functions at its binding sites. Previous studies have demonstrated a link between RNA Pol II and insulation; some Pol II-enriched promoters function as enhancer-blocking elements when these sequences are inserted between the enhancer and promoter (105,106), and promoters with strong Pol II accumulation physically interact with the insulator proteins (107). Consistent with these observations, approximately half of the Mod(mdg4) peaks were on promoters (Figure 3D). It is plausible that Mod(mdg4)-N acts as an insulator by regulating the accumulation of Pol II. To analyze the relationship between Mod(mdg4)-N and promoter-proximal pausing, we first reanalyzed public data for OSC Pol II ChIP (71) using the pausing index (Supplementary Figure S8A). The pausing index is the ratio of the Pol II ChIP-seq density in the region near the TSS (±250 bp) to that in the gene body (70). The higher this index, the greater the pausing observed at the promoter. The promoters with Mod(mdg4)-N had a significantly higher pausing index than all promoters (Supplementary Figure S8B), indicating that Mod(mdg4)-N tends to bind highly to Pol II-pausing promoters. Furthermore, TAS-R is similar to *Invader4* LTR; thus, it contains core promoter sequences, specifically Initiator (Inr) (108) and downstream promoter element (DPE) (109), according to the criteria described in previous research (110) (Supplementary Figure S9A). These results indicate the involvement of the Pol II machinery in the regulation of the subtelomeric enhancer-blocking function of Mod(mdg4)-N.

**Figure 6.**
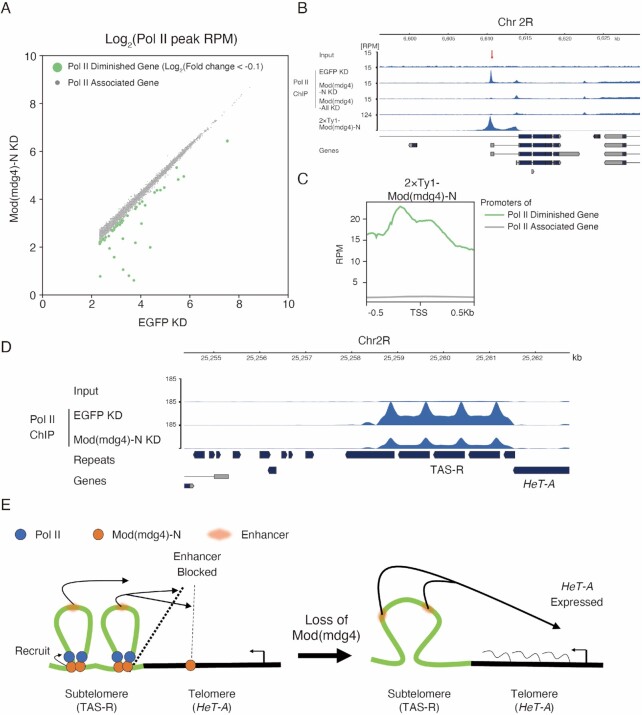
RNA polymerase II is recruited to Mod(mdg4)-N binding sites including subtelomeric TAS-R repeats. (**A**) Scatter plot of maximum peak coverage between EGFP KD (x-axis) and Mod(mdg4)-N KD (y-axis). Both the x-axis and y-axis are shown with log_2_ ratios. Peaks whose maximum values were under 5.0 at RPM in EGFP KD were removed from the analysis. Pol II-diminished genes (green) are defined as log_2_(fold changes) < −0.1, and other genes (Pol II-associated genes) whose Pol II level did not change with the Mod(mdg4)-N KD are shown with gray dots. (**B**) Coverage tracks showing examples of Mod(mdg4)-N localization and Pol II profile with EGFP KD, Mod(mdg4)-N KD, and Mod(mdg4)-all variants KD at the Pol II diminished gene locus (Chr2R:6.60Mb-6.63Mb). The lower panel indicates annotations of the transcripts from Flybase. The red arrow indicates the Pol II peak that diminished with KD of Mod(mdg4)-N or all variants. (**C**) The average profile showing enrichment of Mod(mdg4)-N ChIP-seq intensity around the Pol II peaks that diminished with the Mod(mdg4)-N KD (Pol II diminished genes; green) or the other genes (Pol II associated gene, gray). (**D**) Coverage tracks showing input and Pol II enrichment in the TAS-R regions with the EGFP KD and Mod(mdg4)-N KD. The lower panel indicates annotations of the transcripts from Flybase. (**E**) Summary of this research. Mod(mdg4)-N (orange) binds on both TAS-R and telomeric *HeT-A* retrotransposons. Mod(mdg4)-N represses enhancer activity at both TAS-R and *HeT-A*, although enhancer-blocking activity at TAS-R is stronger. Mod(mdg4)-N recruits Pol II (blue) on the subtelomeric TAS-R repeats, which assists in blocking the subtelomeric enhancer (red). Therefore, *HeT-A* is silenced in the normal state. Upon loss of Mod(mdg4)-N, enhancer-blocking activity is lost, leading to *HeT-A* expression.

We next examined how the Pol II peak changes with the Mod(mdg4)-N KD using a Pol II ChIP-seq analysis. Mod(mdg4)-N bound only to selected promoters; therefore, global changes in the Pol II ChIP-seq signals were not observed with the Mod(mdg4)-N KD. However, some Pol II peaks were significantly diminished (log_2_(fold change) < −0.1) upon Mod(mdg4)-N KD (Figure 6A, B). In contrast, almost no Pol II peaks became more pronounced upon Mod(mdg4)-N KD. We also observed that Mod(mdg4)-N ChIP-seq signals were highly enriched on these ‘Pol II Diminished Genes’ (Figure 6C), indicating that Mod(mdg4)-N had a Pol II-recruiting function at its binding site. Importantly, the TAS-R repeats at the subtelomeres were also associated with Pol II, and the KD of Mod(mdg4)-N resulted in a diminished accumulation of Pol II in this region (Figure 6D). In contrast, changes of Pol II accumulation were not observed at the *HeT-A* sites (Supplementary Figure S8C).

This observation implies that Pol II, regulated by Mod(mdg4)-N, may play a critical role in the enhancer-blocking activity. To test this, we investigated whether the loss of the TAS-R core promoter sequence attenuates its enhancer-blocking activity in the OSC. We designed a reporter in which the *Tj* enhancer acts on the DSCP, which transcribes EGFP:P2A:BlastR, and assessed the *Tj* enhancer-blocking activity of the TAS-R mutant sequences by the EGFP intensity (Supplementary Figure S9B). This reporter vector was integrated into chromatin using the PiggyBac system (Supplementary Figure S9C). To analyze the impact of the Pol II association on the enhancer-blocking activity, we compared two TAS-R sequence mutants for the enhancer reporter assay: the first mutant was a complete deletion of the core promoter sequences, Inr and DPE, from a TAS-R sequence; the second mutant was a replacement of the Inr sequence with a random sequence (Supplementary Figure S9A). Using this reporter assay, we observed the enhancer-blocking activity of the two copies of TAS-R repeats (Supplementary Figure S9D), which is consistent with the live-imaging assay (Figure 5). Both the deletion and replacement of the TAS-R core promoter sequences resulted in an increased EGFP intensity compared with the wild-type TAS-R. This indicates that the enhancer-blocking activity weakened with the core promoter loss or replacement (Supplementary Figure S9D). Thus, Pol II recruitment to the TAS-R repeats can increase the enhancer-blocking activity, supporting the importance of Pol II recruitment to TAS-R by Mod(mdg4)-N. Altogether, we showed that a specific Mod(mdg4) variant represses the *HeT-A* retrotransposon by blocking enhancers in subtelomeric repeats, and the recruitment of Pol II can reinforce this function (Figure 6E).

## DISCUSSION

### Enhancer and insulator function of *Drosophila* subtelomeres

We have demonstrated Mod(mdg4)-N-mediated repression of *HeT-A* retrotransposon (Figures 1, 2), Mod(mdg4)-N binding at telomeric/subtelomeric regions and the existence of enhancer activity in TAS-R (Figures 3, 4), enhancer blocking activity of Mod(mdg4)-N-binding TAS-R and *HeT-A* sequences (Figure 5), and the importance of Pol II association in Mod(mdg4)-N-mediated enhancer-blocking (Figure 6). Based on these results, we propose a model in which the enhancer function of the TAS-R region is blocked by Mod(mdg4)-N, which in turn represses the expression of *HeT-A* at telomeres (Figure 6E).

We observed diminished accumulation of Pol II on TAS-R upon Mod(mdg4)-N depletion and deletion or mutation of core promoter sequences in TAS-R attenuated but did not completely abolish its insulator activity (Supplementary Figure S9). With this observation, we speculate that two mechanisms are involved in the enhancer-blocking function of Mod(mdg4)-N. The first is Pol II recruitment-dependent insulator activity (Figure 6, Supplementary Figure S9). In *Drosophila*, several insulator proteins exist, and some of these insulator proteins, including Mod(mdg4), are enriched near TSSs (89,111). Functionally, some of the insulator-binding promoters work as enhancer-blocking elements when these sequences are inserted between an enhancer and another promoter (105,106). Highly paused promoters are associated with insulator-binding sites (107), indicating that promoters can play an essential role in the insulator-mediated enhancer-blocking. Additionally, it has been proposed that regulation of chromatin conformation is important for enhancer-blocking activity (101,112). In fact, there is an example of the association of enhancer blocking activity with transcriptional regulation at promoters: M1BP is required for both Pol II recruitment and enhancer-blocking activity, and furthermore, transcriptional regulation by M1BP is coupled with chromatin conformation change (113,114). Thus, previous studies indicate a link between transcriptional machinery and enhancer-blocking activity. Taking these into account, Mod(mdg4)-N might cause chromatin conformation change mediated by transcriptional regulation at promoter sequences, which leads to enhancer-blocking activity at TAS-R. The second is the biochemical nature of insulator proteins. Mod(mdg4) has a BTB/POZ domain at its N-terminal common region, which can form multimers with both itself and a BTB/POZ domain of Trl (Trithorax-like, a.k.a. GAGA Factor) (115,116). Therefore, the homophilic nature of the Mod(mdg4) BTB/POZ domain contributes to enhancer-blocking by dynamic loop formation between different Mod(mdg4)-N associated sites (101,104).

Overall, our results indicate that Mod(mdg4)-N is necessary for *HeT-A* repression by blocking subtelomeric enhancers. Interestingly, the enhancer-like function of subtelomeres is also reported in human, and transcription of TERRA (telomeric repeat-containing RNA) is promoted by insulator protein CTCF despite telomeric sequences in human and fly being completely different (14,117). This observation shows an evolutionarily conserved principle of enhancer-like functions at subtelomeres being modulated by insulator proteins. What drives this subtelomeric convergent evolution is still an open question.

### Specificity of Mod(mdg4)-N mediated enhancer-blocking activity at subtelomeric TAS-R and *HeT-A* repeats

We showed that Mod(mdg4)-N regulates gene expression almost exclusively at subtelomere/telomere regions (Figure 4B-C). This result suggests that Mod(mdg4)-N does not regulate its associated genes, but functions in a context-dependent manner, limiting its impact to telomeric regions. Also, since TAS-R repeats are the source of enhancer-blocking activity, the role of Mod(mdg4)-N in regulating telomeric transcription is limited to the telomeric sites with TAS-R repeats.

For Mod(mdg4)-N to block the enhancer, it is essential to have Mod(mdg4) located at certain genomic regions containing both enhancer and downstream promoter. We also found that Mod(mdg4)-N is responsible for recruiting Pol II at only limited genomic regions (Figure 6A). Additionally, we showed that Mod(mod4)-N-mediated Pol II association within TAS-R plays an important role in enhancing the regulation of downstream genes (Supplementary Figure S9). Therefore, the requirement of Pol II association sites along with enhancer sites limits the enhancer-blocking activity of Mod(mdg4)-N to certain genomic locations. Another important requirement is the tandem-repeating nature of the Mod(mdg4)-N association sites. We demonstrated that multiple copies of TAS-R sites, capable of recruiting Mod(mdg4)-N, can induce an insulator function significantly stronger than that of the single copy of TAS-R (Figure 5B–F).

In summary, the key factors for inducing a strong enhancer-blocking effect, specifically at the TAS-R regions, are both the Pol II association sites within the TAS-R, capable of recruiting Pol II with the Mod(mdg4)-N association, and the highly repetitive nature of the TAS-R sequences. Overall, these results suggest that regulation on TAS-R is the major mechanism of Mod(mdg4)-mediated *HeT-A* expression, and Mod(mdg4)-N-binding *HeT-A* sequences are functional but have a minor contribution to *HeT-A* transcriptional regulation. This enhancer-blocking activity of Mod(mdg4)-N is a novel mechanism regulating *HeT-A* expression, ensuring genomic stability in *Drosophila*.

### Transcriptional regulation of telomeric retrotransposons

We showed that Mod(mdg4)-N represses *HeT-A* both *in vivo* and *in vitro* (Figures 1,2). Although Mod(mdg4) is ubiquitously expressed in the ovary (Figure 2A–E), Mod(mdg4)-N is especially important for the regulation of *HeT-A* in follicle cells where the PIWI–piRNA pathway does not target *HeT-A*. This result indicates that the Mod(mdg4)-mediated repression machinery is a compensatory mechanism for telomeric retrotransposon regulation. The PIWI–piRNA pathway directs heterochromatin formation on telomeres, and dysfunction of this pathway leads to a dramatic increase in *HeT-A* expression in the germline (38–41). Therefore, it is likely that even without Mod(mdg4)-N, the PIWI–piRNA pathway is strong enough to repress *HeT-A* expression in germline cells. The chromatin states of subtelomeres and telomeres are reported to differ among cell types, especially between germ cells and somatic cells (118). It is also possible that different chromatin states at the subtelomeres and telomeres prevent the binding of Mod(mdg4)-N in certain cell types.

We showed that subtelomeric enhancer activity is regulated by Mod(mdg4)-N associated with the subtelomeric region. This complex regulation may be due to the dynamic regulation of *HeT-A* expression in somatic cells, to enable expression of *HeT-A* under specific biological conditions. Also, this study highlights the transcriptional regulation of *HeT-A* associated with TAS-R, which harbors enhancer activity. *HeT-A* associated with TAS-L, without enhancer activity or Mod(mdg4) association, may be regulated by another mechanism. Multiple mechanisms regulating *HeT-A* are reported in a cell-type specific manner (33,36–46,119,120). We believe that future studies will elucidate interactions between these regulation mechanisms, and the biological significance of *HeT-A* being regulated by different mechanisms.

## DATA AVAILABILITY

GSE (ChIP-seq, RNA-seq): GSE176196.

Github (python Script): https://doi.org/10.5281/zenodo.7232511.

FACS rawdata: http://flowrepository.org/id/FR-FCM-Z5Y3.

## Supplementary Material

gkac1034_Supplemental_Files

## Notes

Present address: Shu Kondo, Department of Biological Science and Technology, Faculty of Advanced Engineering, Tokyo University of Science, Tokyo 125-8585, Japan.
