# Supplementary material for: Mod(mdg4) variants repress telomeric retrotransposon HeT-A by blocking subtelomeric enhancers
Source: Nucleic Acids Res. 2022 Nov 14;50(20):11580–99. doi: 10.1093/nar/gkac1034 (PMC9723646; doi:10.1093/nar/gkac1034)
Supplement: gkac1034_Supplemental_Files [file gkac1034_supplemental_files.zip › Supplemental_Information.pdf]

Supplemental figure legends

Fig. S1 *HeT-A* is not repressed by heterochromatin-dependent mechanisms

A. Relative expression calculated from RNA-seq reads mapped to *mdg1* and *HeT-A* upon EGFP (control), piwi, or H1 KD. Read counts are normalized by reads per million (RPM) and the expression level at EGFP KD as 1.

B. Coverage tracks showing results of H3K9me3 ChIP-seq (blue) and its input (black line) mapped onto the consensus sequence of *mdg1* and *HeT-A* from repbase. Coverages are normalized by counts per mapped million reads.

C. Bar plot showing relative expression of each Mod(mdg4) variant in OSC as revealed by EGFP-KD RNA-seq. Variants G and L have no unique sequence and are therefore excluded. The names of variants tested in the siRNA KD screen (Fig. 1E) are indicated with orange.

D-F. Western blotting shows the specificity of the antibodies used in this study. Antibodies against Mod(mdg4) variant N (D), Mod(mdg4) common region (E) and HeT-A Gag (F) were used. We checked the specificity of antibody by KD of endogenous Mod(mdg4) in OSC (D,E). Because HeT-A Gag protein is not expressed in normal conditions, we checked the specificity of antibody by comparing no vector control, Myc-EGFP or Myc-EGFP-Gag protein overexpression (F) in OSC. Tubulin is detected as a loading control for each experiment. Note that left and right panel uses the same lysate for the western blot, and therefore, there is only one tubulin control for these two gels. Molecular weight (MW) is indicated at the left of each image.

Fig. S2 Source images of Western blots

A-C. Source images of western blots of Fig. 1F. Experiments were performed with indicated antibodies; (A) Het-A Gag antibody, (B,C) Mod(mdg4)-N antibody (left), Mod (mdg4) common region antibody (right), beta-tubulin antibody (lower). The letters above images indicate experiment samples: (1) Myc-EGFP-Het-A Gag overexpression (o/e), (2) Myc-EGFP o/e, (3) EGFP KD, (4) Mod(mdg4)-N KD, (5) Mod(mdg4)-All KD. (C) is the long exposure of (B). Myc-EGFP-Het-A Gag o/e samples were used as positive controls.

D-F. Source images of western blots of Fig. 2A. Experiments were performed with indicated antibodies; (D-E) Mod (mdg4) common region antibody(left), Het-A Gag antibody (right), beta-tubulin antibody (lower). (F) Het-A Gag antibody (left), Mod (mdg4)-N antibody (right), beta-tubulin antibody (lower). The letters above images indicate experiment samples: (1) Myc-EGFP-Het-A Gag overexpression (o/e) 1/50 dilution, (2) Myc-EGFP o/e 1/50 dilution, (3) Wildtype (OregonR), (4) Mod(mdg4)$^{RN1\text{-}2/RN1\text{-}4}$. (E) is the long exposure of (D). Myc-EGFP-Het-A Gag o/e samples were used as positive controls.

Fig. S3 Genes regulated in Mod(mdg4)-N mutants

A. Fly strain (*Mod(mdg4) RN1-2* and *RN1-4*) used in this study. The result of sanger sequencing of *Mod(mdg4) RN1-2* heterozygote is shown in upper panel. Their sequences are indicated below. *Mod(mdg4) RN1-2* and *RN1-4* have two or four base pairs missing from their variant-specific exon.

B. Western blotting showing protein levels of HeT-A Gag, Mod(mdg4)-N, total Mod(mdg4) and tubulin (loading control) in ovaries of +/+ wildtype (OregonR) and homozygous *Mod(mdg4)* $^{RN1\text{-}2/RN1\text{-}2}$(left) or *Mod(mdg4)*$^{RN1\text{-}4/RN1\text{-}4}$(right) mutant. Molecular weight (MW) is indicated at the left of each image.

C. Bar plot showing *HeT-A* expression measured by RT-qPCR. y-axis is normalized by *RP49* (n=6 for WT or *Mod(mdg4)*$^{RN1\text{-}2/RN1\text{-}2}$ and n=5 for *Mod(mdg4)*$^{RN1\text{-}4/RN1\text{-}4}$). Average of WT ovary is normalized as 1. Each dot indicates values obtained from different experiments.

D. Bar plot showing results of crossbreeding experiments with indicated genotypes of female flies and wild-type (OregonR) male flies. RN1-2 and RN1-4 indicate lines bearing Mod(mdg4)-N variant-specific mutants (Fig. S3A). The y-axis represents the eggs per fly for 3 h. Each dot indicates the value obtained from different experiments. Asterisks indicate $p < 0.05$ in the two-sided t-test.

E. Stereomicroscopic images of ovaries with the indicated genotypes (left: *Mod(mdg4)*$^{RN1\text{-}2/RN1\text{-}4}$, right: *Mod(mdg4)*$^{RN1\text{-}4/+}$). The white bar indicates 2 mm.

F. Immunostaining images of egg chambers from *Mod(mdg4)*$^{RN1\text{-}2/RN1\text{-}4}$ (left) and *Mod(mdg4)*$^{RN1\text{-}4/+}$ (right) mutant flies. Ovaries were stained with Orb (magenta) and DAPI (blue). White bars indicate 100 μm.

G. Confocal images of egg chambers from *Mod(mdg4)*$^{RN1\text{-}2/RN1\text{-}4}$ (left) and *Mod(mdg4)*$^{RN1\text{-}4/+}$ (right) mutant flies in a low-power field (10×). The upper panels are images of DAPI staining, and the lower panels are bright-field images. Black arrows indicate multiple late-stage egg chambers that remain in the ovaries.

H. MA plot of TPM values ($\log_{10}$ scale) for mRNA in the Mod(mdg4)$^{RN1\text{-}2/RN1\text{-}2}$ versus WT (y^1w^{1118}) ovaries (n=3 for each condition). Differentially expressed genes (DEGs) are shown as red dots.

I. Gene ontology analysis of up-regulated (upper panel) and down-regulated (lower panel) genes in Mod(mdg4)$^{RN1\text{-}2/RN1\text{-}2}$ mutant ovaries. Bar plots indicates -$\log_{10}$(FDR) value of each gene ontology.

Fig S4 Mod(mdg4) is not associated with TAS-L repeats

A. Western blotting using Ty1-tag antibody showing exogenous expression of 2×Ty1-tagged Mod(mdg4)-AF, N, V or T in OSC. Tubulin was used for loading control. Molecular weight (MW) is indicated at the left of each image.

B. Logos showing clear enrichment around ChIP-seq peaks of Mod(mdg4)-V and -AF. These motifs are identified by de novo motif discovery analysis MEME (Multiple Em for Motif Elicitation).

C. Sequence logos of motif of Su(Hw) from OntheFly (122) and the enriched motif of 2xTy1-Mod(mdg4)-T ChIP-seq peaks (this sequence logo is the same as Fig.3E).

D. Coverage panel showing ChIP-seq of Mod(mdg4)-N and -T on *TAS-L* region from dm6. Coverage of each ChIP-seq experiment is shown with blue. The y-axis is normalized with RPM.

Fig. S5 TAS-L is not a functional regulatory element

A. Coverage panel showing STARR-seq signal (purple) on the consensus sequence of *HeT-A* from repbase. The y-axis is normalized with RPM. Below coverage panel of *HeT-A*, the reading frame of HeT-A Gag protein is shown in orange, and the black arrow indicates the *HeT-A* promoter. This data is reanalysis from STARR-seq results of Arnold CD et al., 2013.

B. Coverage panel showing STARR-seq signal (purple) for OSC and its input on TAS-L region from dm6. The y-axis is normalized with RPM. This data is a reanalysis from STARR-seq results of Arnold CD et al., 2013.

Fig. S6 Sequences used for live-imaging assay

Coverage plot showing sequences used for live-imaging of enhancer-blocking assay. chr4: 1286805-1287803 (dm6) region is referred to as *HeT-A*, and chr2R: 25259656-25259137 (dm6) is referred to as TAS-R in this study. Red bars indicate the sequences used for enhancer-blocking assay. Mod(mdg4)-N ChIP-seq coverage (blue) and Repeatmasker annotations of repetitive sequences are indicated. Note that there are some mutations in cloned sequences compared with reference and precise sequences used for the assay are written in Supplemental Table 2.

Fig. S7 Mod(mdg4)-N directly regulates the enhancer-blocking activity of TAS-R

A. Schematic representation of plasmid used for the dual-luciferase assay. In the Nanoluc (Nluc) plasmid, Nluc is transcribed from *Drosophila* synthetic core promoter (DSCP). In TAS-R + TAS-R plasmid, TAS-R sequences (Fig. S6, Supplemental Table 2) are inserted at both sides of the *Tj* enhancer. In the Firefly luciferase (Fluc) plasmid, Fluc is transcribed from the DSCP.

B. Bar plot showing the result of the dual-luciferase assay. Values on x-axis are normalized relative to the average of the control plasmid. Each dot indicates values obtained from different experiments (n=4). Asterisk indicates $p<0.05$ in the two-sided t-test.

C. Schematic representation of plasmid used for the dual-luciferase assay. In the TAS-R + TAS-R plasmid, the *Tj* enhancer is surrounded by two copies of subtelomeric TAS-R sequences. Nanoluc is transcribed from the DSCP. In the Firefly luciferase (Fluc) plasmid, Fluc is transcribed from the DSCP.

D. Bar plot showing the result of the dual-luciferase assay. Values on x-axis are normalized relative to the average of EGFP KD. Each dot indicates values obtained from different experiments (n=4). Asterisk indicates $p<0.05$ in the two-sided t-test.

Fig. S8 Calculation of pausing index at Mod(mdg4)-N association sites

A. Schematic diagram showing how pausing index is calculated. Pausing index is the value obtained from dividing the number of reads/kb in the promoter region (TSS ±250 bp) by the number of reads/kb in the gene body (TSS +500 bp ~ TTS -250 bp) region.

B. Cumulative distribution plot showing pausing index of all promoters (orange) and Mod(mdg4)-N bound promoters (blue) from reanalysis from Sienski G et al, 2011. (Kolmogorov-Smirnov test $p= 2.22 \times 10^{-11}$). The x-axis is $\log_2$ ratio.

C. Coverage tracks showing input and Pol II enrichment on *HeT-A* regions upon EGFP KD or Mod(mdg4)-N KD. Below the coverage panel, the reading frame of HeT-A Gag protein is shown in orange, and the black arrow indicates the *HeT-A* promoter.

Fig. S9 Enhancer blocking function depends on Pol II recruiting machinery

A. TAS-R sequence and its mutation used for experiment. Initiator (Inr) sequence (green) and downstream promoter element (DPE) sequence (blue) are indicated. Two mutants (Promoter deletion and Inr mutant) are generated from this sequence as shown (red).

B. Schematic representation of reporter gene used for assay. *Tj* enhancer is located downstream of *Drosophila* synthetic core promoter-EGFP:P2A:Blastcidin-S deaminase gene. Actin5C promoter-mCherry:P2A:Puromycin N-acetyl transferase provides a selective marker. This plasmid is inserted into the genome with *piggyBac* transposase.

C. Experimental design of assay. Reporter gene is integrated into the genome with PiggyBac transposase. Then, successfully integrated reporter is selected with puromycin. After 13 days of selection, mCherry-positive cells are sorted and EGFP-intensity is measured with FACS.

D. Violin plot showing the results of normalized EGFP intensity of no insertion control, downstream TAS-R with wild-type TAS-R insertion, promoter deletion mutant or initiator mutant, from left to right. Mean and median values of EGFP intensity are shown below. The y-axis is $\log_{10}$ ratio. An asterisk (*) indicates the hypothesis that these two distributions are from same population is rejected at $p = 0.01$ significant level with Brunner-Munzel test.

Supplemental Video 1
Live imaging of MS2-yellow-sna shadow enhancer reporter with no insertion of insulators (WT), single insertion of *gypsy* insulator, *HeT-A* sequence, or TAS-R sequence and double insertion of TAS-R sequence from left to right during nc14. The maximum projected images of MCP-GFP and His2Av-mRFP are shown in green and red, respectively. Images are oriented with ventral view facing up.

Supplemental Video 2
Live imaging of MS2-yellow-sna shadow enhancer reporter with no insertion of insulators (left) and double insertion of *HeT-A* sequence (right) during nc14. The maximum projected images of MCP-GFP and His2Av-mRFP are shown in green and red, respectively. Images are oriented with ventral view facing up.

Supplemental Table 1
Primers and siRNAs used in this study

Supplemental Table 2
Sequences used for reporter assays

Supplemental Table 3
The list of differentially expressed genes in Mod(mdg4)-N mutant compared with Wild-type(y^1 w^{1118}) ovaries.

Supplemental Table 4
RNA-FISH probes used in this study

Figure S1

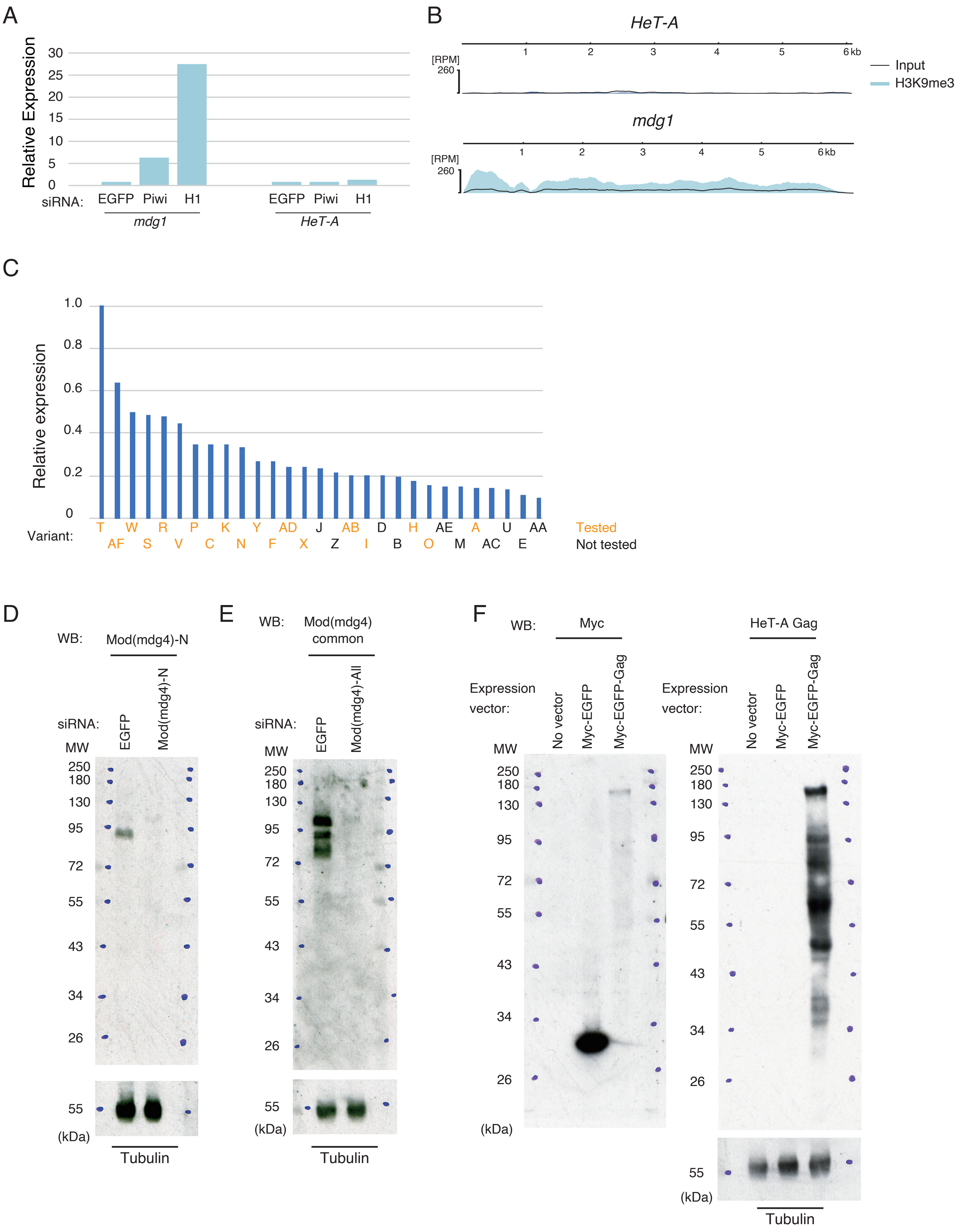
A
Relative Expression
siRNA: EGFP Piwi H1 EGFP Piwi H1
mdg1
HeT-A
B
HeT-A
mdg1
[RPM] 260
Input
H3K9me3
C
Relative expression
Variant:
T W R P K Y AD J AB D H AE A U AA
AF S V C N F X Z I B O M AC E
Tested
Not tested
D
WB: Mod(mdg4)-N
siRNA: EGFP Mod(mdg4)-N
MW
(kDa)
Tubulin
E
WB: Mod(mdg4) common
siRNA: EGFP Mod(mdg4)-All
MW
(kDa)
Tubulin
F
WB: Myc
HeT-A Gag
Expression vector:
No vector
Myc-EGFP
Myc-EGFP-Gag
MW
(kDa)
Tubulin

Figure S2

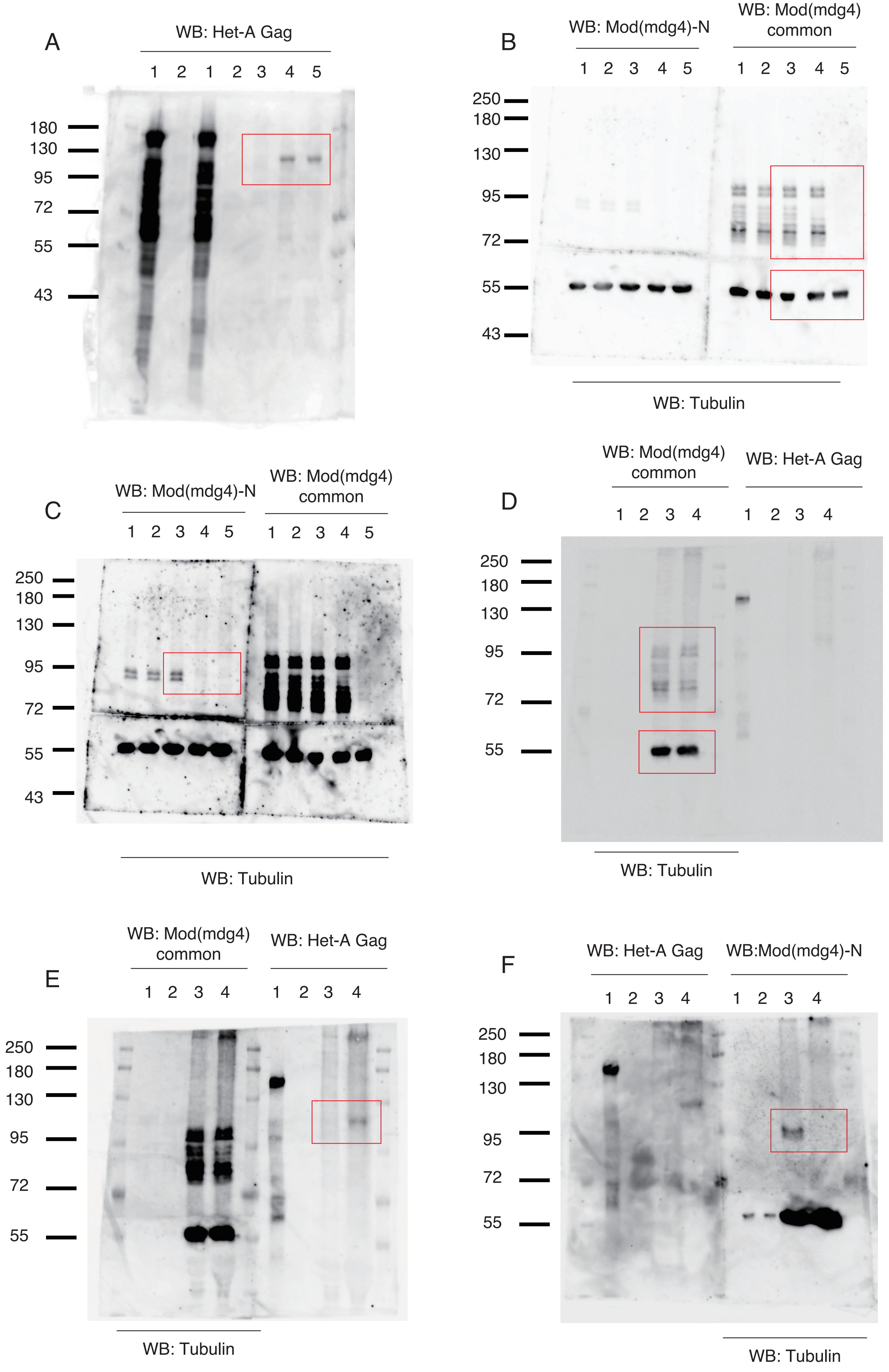
A
WB: Het-A Gag
1 2 1 2 3 4 5
180
130
95
72
55
43
B
WB: Mod(mdg4)-N
WB: Mod(mdg4) common
1 2 3 4 5
1 2 3 4 5
250
180
130
95
72
55
43
WB: Tubulin
C
WB: Mod(mdg4)-N
WB: Mod(mdg4) common
1 2 3 4 5
1 2 3 4 5
250
180
130
95
72
55
43
WB: Tubulin
D
WB: Mod(mdg4) common
WB: Het-A Gag
1 2 3 4
1 2 3 4
250
180
130
95
72
55
WB: Tubulin
E
WB: Mod(mdg4) common
WB: Het-A Gag
1 2 3 4
1 2 3 4
250
180
130
95
72
55
WB: Tubulin
F
WB: Het-A Gag
WB:Mod(mdg4)-N
1 2 3 4
1 2 3 4
250
180
130
95
72
55
WB: Tubulin

Figure S3

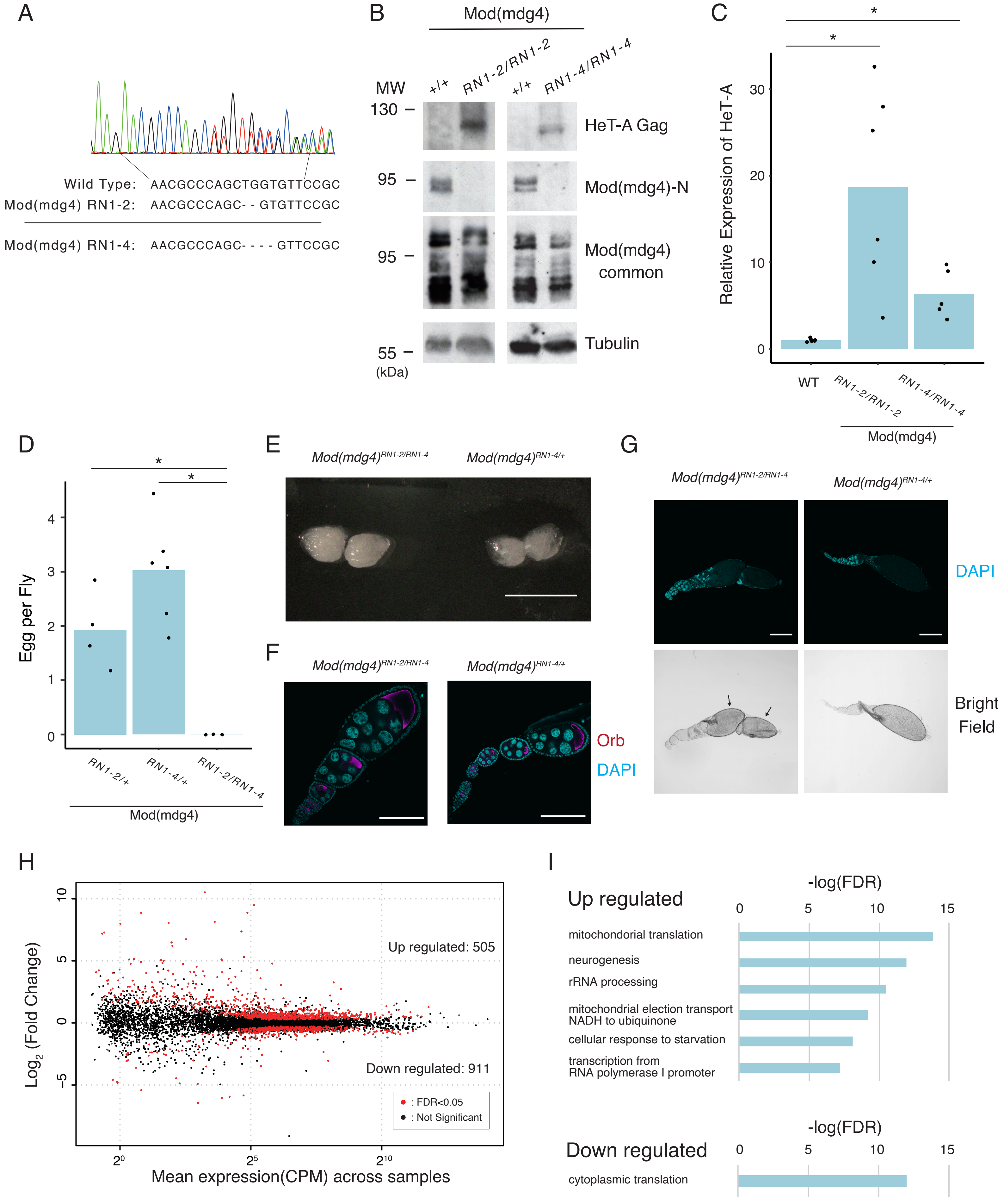
A
Wild Type: AACGCCCAGCTGGTGTTCCGC
Mod(mdg4) RN1-2: AACGCCCAGC- - GTGTTCCGC
Mod(mdg4) RN1-4: AACGCCCAGC- - - - GTTCCGC
B
Mod(mdg4)
MW
+/+
RN1-2/RN1-2
+/+
RN1-4/RN1-4
130
95
95
55
(kDa)
HeT-A Gag
Mod(mdg4)-N
Mod(mdg4) common
Tubulin
C
*
*
Relative Expression of HeT-A
30
20
10
0
WT
RN1-2/RN1-2
RN1-4/RN1-4
Mod(mdg4)
D
*
*
Egg per Fly
4
3
2
1
0
RN1-2/+
RN1-4/+
RN1-2/RN1-4
Mod(mdg4)
E
Mod(mdg4)RN1-2/RN1-4
Mod(mdg4)RN1-4/+
F
Mod(mdg4)RN1-2/RN1-4
Mod(mdg4)RN1-4/+
Orb
DAPI
G
Mod(mdg4)RN1-2/RN1-4
Mod(mdg4)RN1-4/+
DAPI
Bright Field
H
Log2 (Fold Change)
10
5
0
-5
Up regulated: 505
Down regulated: 911
: FDR<0.05
: Not Significant
2^0
2^5
2^10
Mean expression(CPM) across samples
I
Up regulated
-log(FDR)
0
5
10
15
mitochondorial translation
neurogenesis
rRNA processing
mitochondrial election transport NADH to ubiquinone
cellular response to starvation
transcription from RNA polymerase I promoter
Down regulated
-log(FDR)
0
5
10
15
cytoplasmic translation

Figure S4

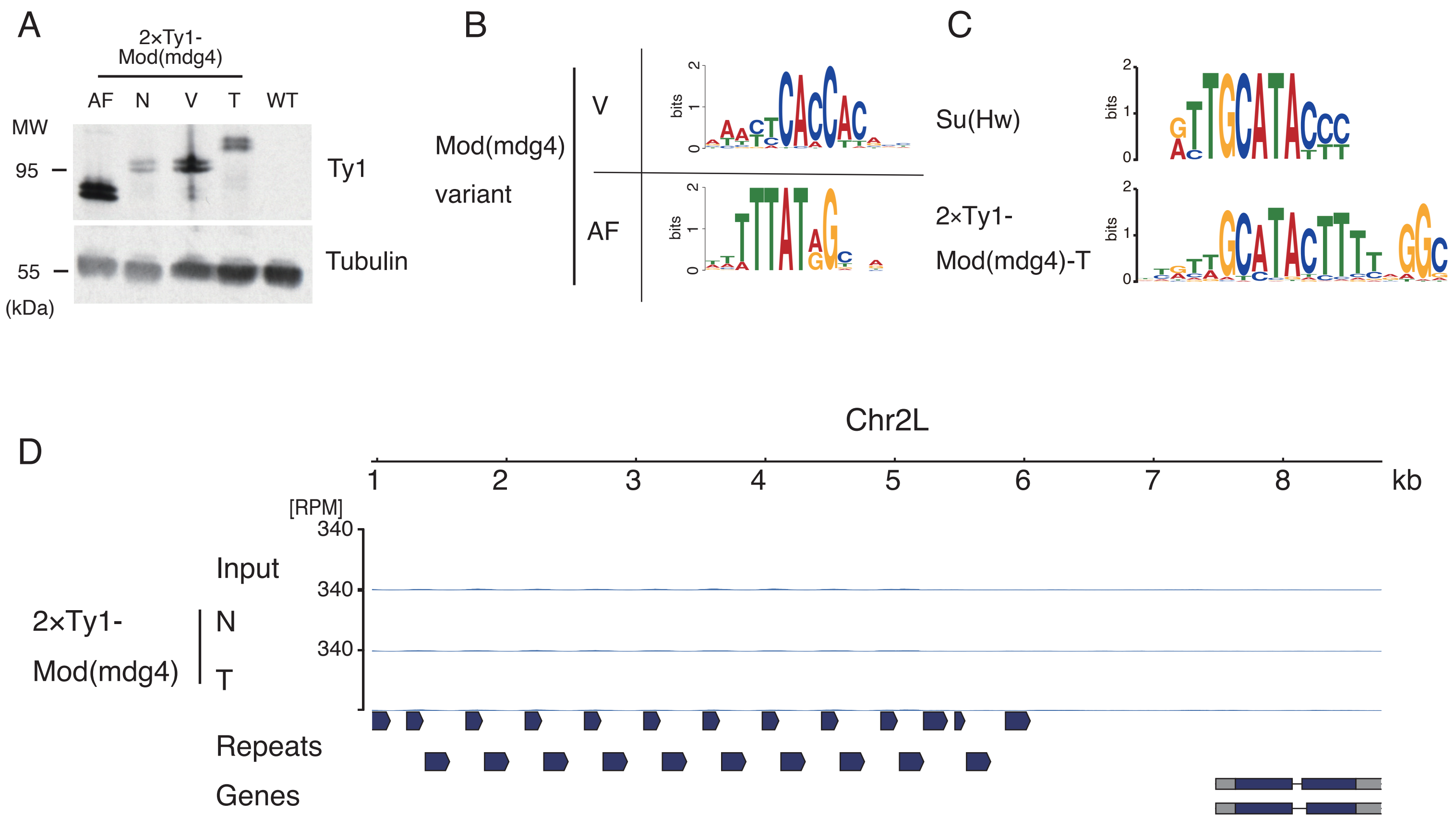

A
2×Ty1-
Mod(mdg4)
AF
N
V
T
WT
MW
95
55
(kDa)
Ty1
Tubulin
B
Mod(mdg4)
variant
V
AF
bits
C
Su(Hw)
2×Ty1-
Mod(mdg4)-T
bits
D
Chr2L
1
2
3
4
5
6
7
8
kb
[RPM]
340
340
340
Input
2×Ty1-
Mod(mdg4)
N
T
Repeats
Genes

Figure S5

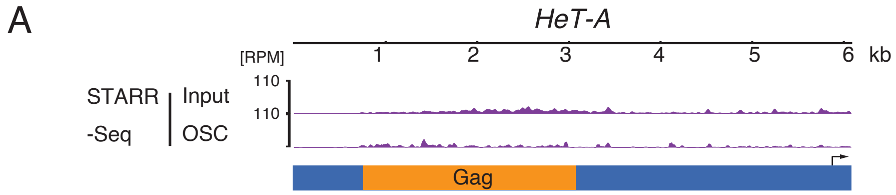

A
HeT-A
[RPM]
1
2
3
4
5
6
kb
110
110
STARR
-Seq
Input
OSC
Gag

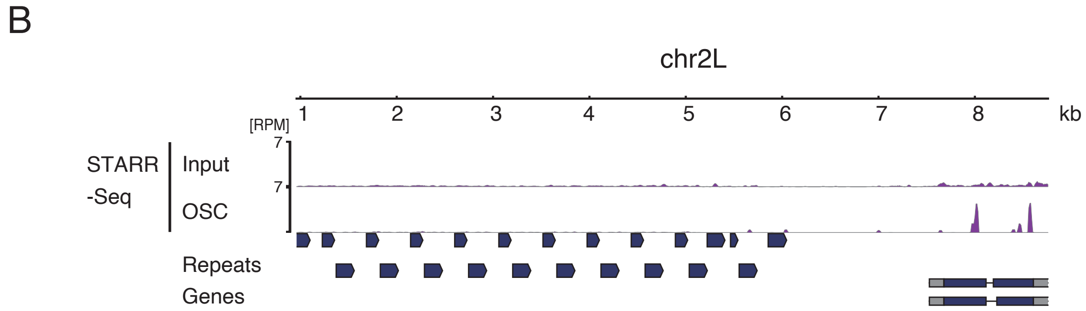

B
chr2L
1
2
3
4
5
6
7
8
kb
[RPM]
7
7
STARR
-Seq
Input
OSC
Repeats
Genes

Figure S6

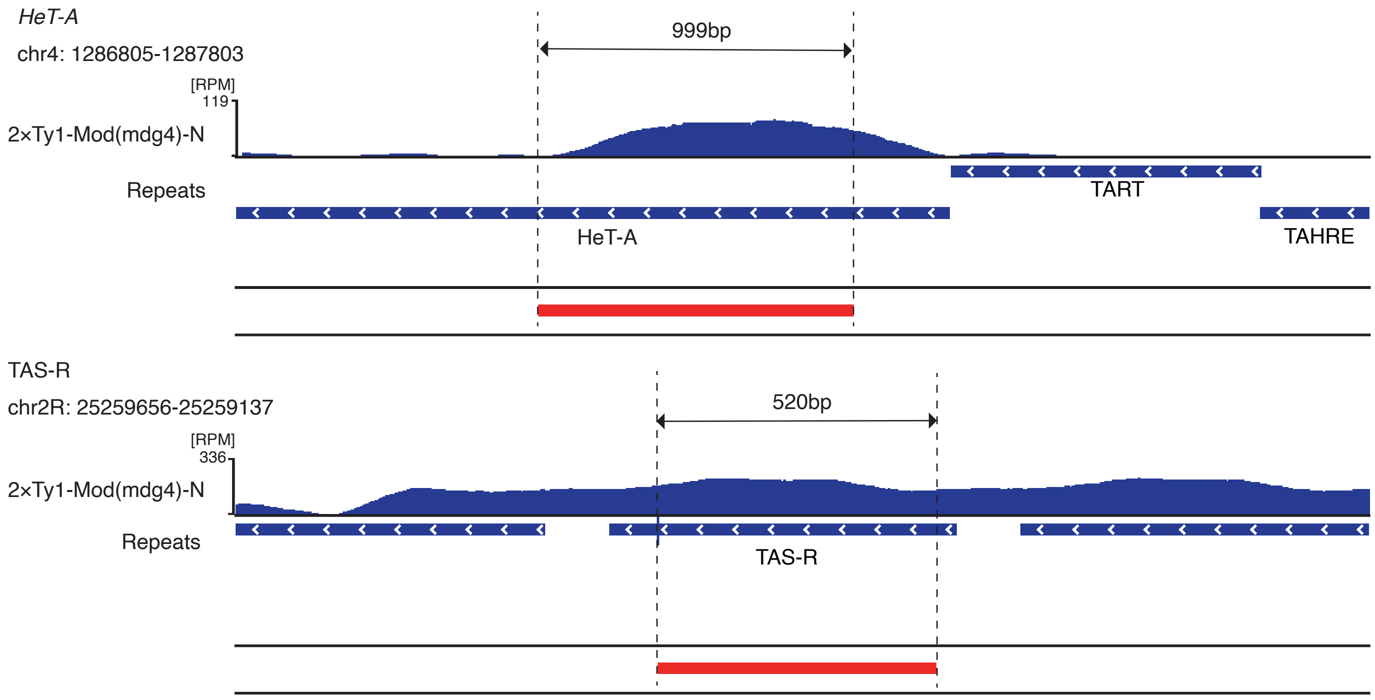
HeT-A
chr4: 1286805-1287803
999bp
[RPM]
119
2×Ty1-Mod(mdg4)-N
Repeats
TART
HeT-A
TAHRE
TAS-R
chr2R: 25259656-25259137
520bp
[RPM]
336
2×Ty1-Mod(mdg4)-N
Repeats
TAS-R

Figure S7

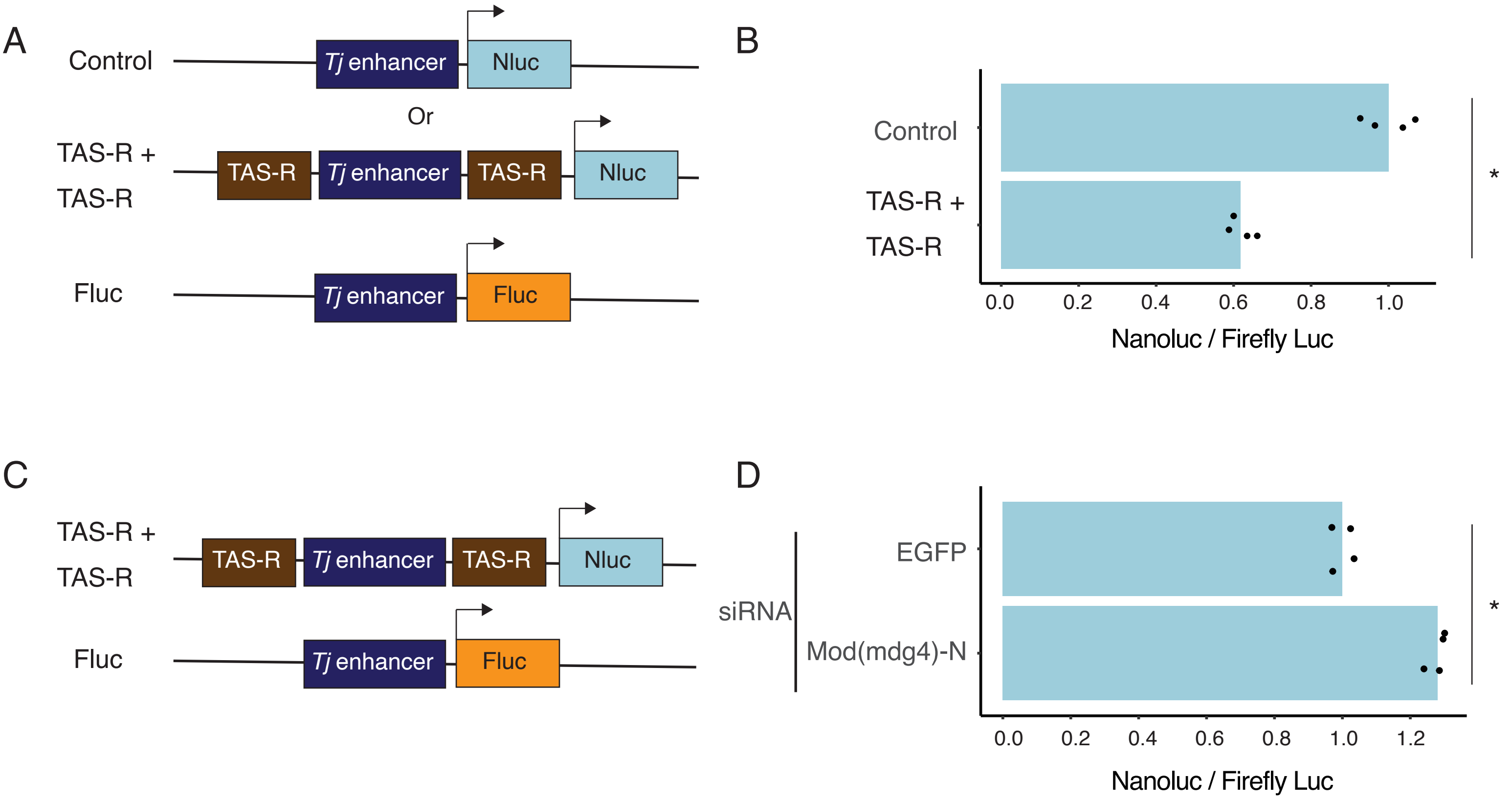
A
Control
Tj enhancer
Nluc
Or
TAS-R +
TAS-R
TAS-R
Tj enhancer
TAS-R
Nluc
Fluc
Tj enhancer
Fluc
B
Control
TAS-R +
TAS-R
*
0.0
0.2
0.4
0.6
0.8
1.0
Nanoluc / Firefly Luc
C
TAS-R +
TAS-R
TAS-R
Tj enhancer
TAS-R
Nluc
Fluc
Tj enhancer
Fluc
D
siRNA
EGFP
Mod(mdg4)-N
*
0.0
0.2
0.4
0.6
0.8
1.0
1.2
Nanoluc / Firefly Luc

Figure S8

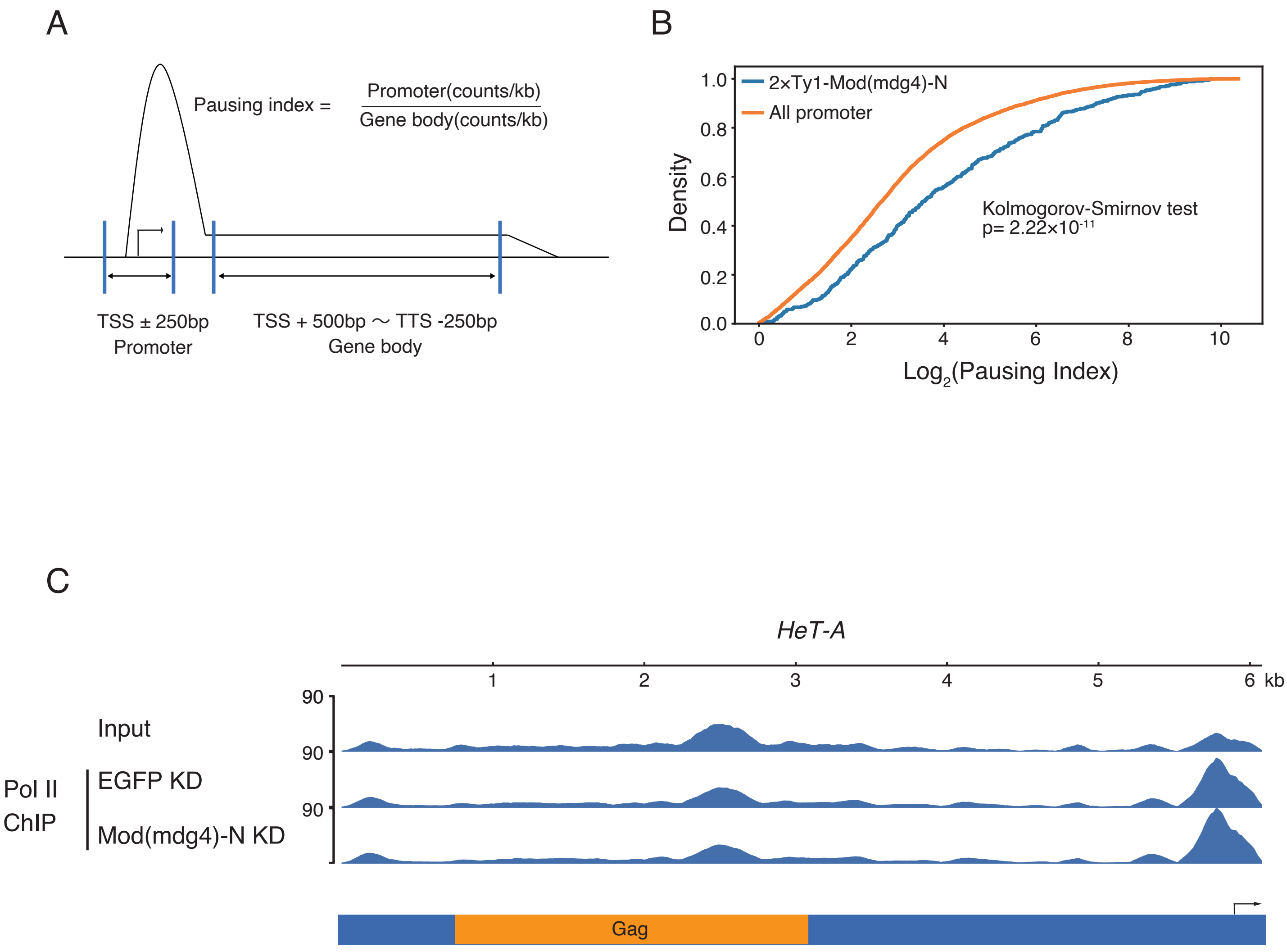
A
Pausing index =
Promoter(counts/kb)
Gene body(counts/kb)
TSS ± 250bp
Promoter
TSS + 500bp ~ TTS -250bp
Gene body
B
2×Ty1-Mod(mdg4)-N
All promoter
Density
Kolmogorov-Smirnov test
p= 2.22×10-11
Log2(Pausing Index)
C
HeT-A
kb
Input
Pol II
ChIP
EGFP KD
Mod(mdg4)-N KD
Gag

Figure S9

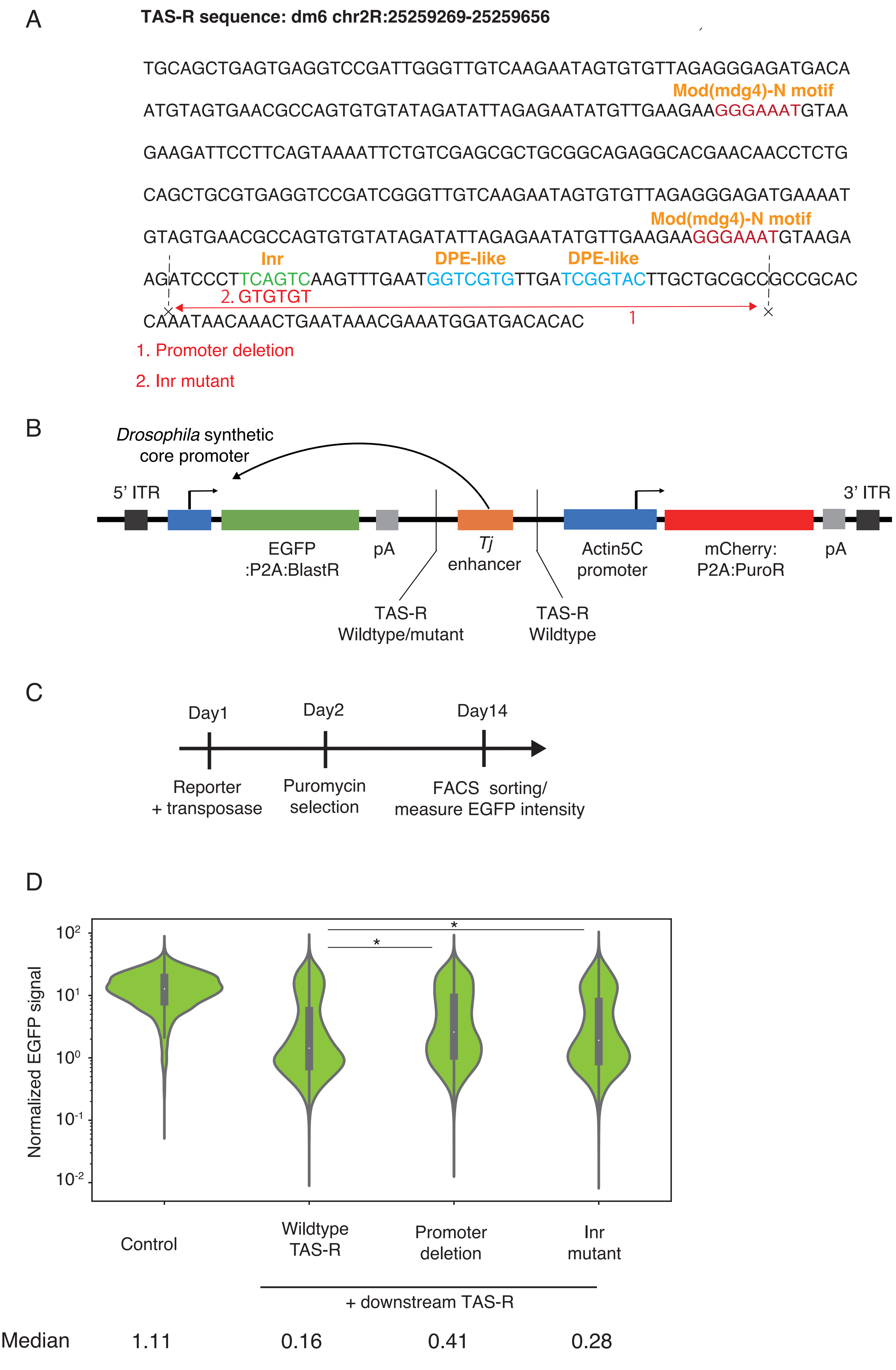
A
TAS-R sequence: dm6 chr2R:25259269-25259656
TGCAGCTGAGTGAGGTCCGATTGGGTTGTCAAGAATAGTGTGTTAGAGGGAGATGACA
Mod(mdg4)-N motif
ATGTAGTGAACGCCAGTGTGTATAGATATTAGAGAATATGTTGAAGAAGGGAAATGTAA
GAAGATTCCTTCAGTAAAATTCTGTCGAGCGCTGCGGCAGAGGCACGAACAACCTCTG
CAGCTGCGTGAGGTCCGATCGGGTTGTCAAGAATAGTGTGTTAGAGGGAGATGAAAAT
Mod(mdg4)-N motif
GTAGTGAACGCCAGTGTGTATAGATATTAGAGAATATGTTGAAGAAGGGAAATGTAAGA
Inr
DPE-like
DPE-like
AGATCCCTTCAGTCAAGTTTGAATGGTCGTGTTGATCGGTACTTGCTGCGCCGCCGCAC
2. GTGTGT
CAAATAACAAACTGAATAAACGAAATGGATGACACAC
1
1. Promoter deletion
2. Inr mutant
B
Drosophila synthetic core promoter
5' ITR
3' ITR
EGFP :P2A:BlastR
pA
Tj enhancer
Actin5C promoter
mCherry: P2A:PuroR
pA
TAS-R Wildtype/mutant
TAS-R Wildtype
C
Day1
Day2
Day14
Reporter + transposase
Puromycin selection
FACS sorting/ measure EGFP intensity
D
Normalized EGFP signal
10^2
10^1
10^0
10^-1
10^-2
*
*
Control
Wildtype TAS-R
Promoter deletion
Inr mutant
+ downstream TAS-R
Median
1.11
0.16
0.41
0.28
